# Chitosan-Based Active Packaging Films Incorporating *Terminalia catappa* Leaf Extract and Zinc Oxide Precursors for Sustainable Food Packaging

**DOI:** 10.3390/polym18080928

**Published:** 2026-04-10

**Authors:** Prem Thongchai, Paitoon Wannapasit, Kulyada Teerasirida

**Affiliations:** Faculty of Agro-Industry, Chiang Mai University, Chiang Mai 50200, Thailand; paitoon.wannapasit@gmail.com (P.W.); kulyada.t@cmu.ac.th (K.T.)

**Keywords:** *Terminalia catappa* leaves, microwave-assisted extraction, zinc oxide, hydrothermal synthesis, active packaging, chitosan film, antioxidant activity, antimicrobial packaging

## Abstract

Chitosan-based active films containing microwave-extracted *Terminalia catappa* leaf extract (TE) and hydrothermally synthesised zinc oxide were developed and characterised. The selected extraction condition (440 W, 20 min, followed by freeze drying) gave 29.5% extract recovery and a total phenolic content of 639.5 mg GAE/g extract. Structural analyses showed that the original crystalline ZnO phase was no longer detectable after film formation under acidic casting conditions, whereas zinc remained present in the film matrix, indicating acid-mediated dissolution and/or structural transformation during casting. Zinc-containing films exhibited higher tensile strength (up to 36.0 MPa), increased glass transition temperature (up to 122.9 °C), and reduced moisture content and water vapour transmission. TE contributed antioxidant activity and light-shielding properties, with antioxidant capacity reaching 22.1 mg Trolox/g film. Films containing ≥0.2% initial ZnO also showed disc-diffusion antimicrobial activity against *Escherichia coli* (up to 22.7 mm) and *Staphylococcus aureus* (up to 20.7 mm). A preliminary 7-day banana-wrapping study further suggested that intermediate formulations containing 0.1–0.2% TE and 0.2–0.3% initial ZnO provided a useful balance among mechanical performance, optical properties, antimicrobial activity, and visual preservation. Overall, zinc–polyphenol–chitosan interactions played an important role in governing film structure and functionality.

## 1. Introduction

The global food industry faces increasing pressure to develop sustainable packaging alternatives that address environmental concerns while ensuring food safety and reducing food waste [1,2,3]. Active packaging systems have emerged as a promising approach by incorporating bioactive compounds that interact with food products or their surrounding environment to maintain quality and enhance safety while supporting more sustainable consumption patterns [4,5,6]. Among natural biopolymers, chitosan has attracted considerable attention because of its biodegradability, film-forming ability, and inherent antimicrobial activity [7,8,9,10]. Chitosan also possesses a distinctive chemical structure rich in free primary amino (–NH_2_) and hydroxyl (–OH) groups. These functional groups not only participate in electrostatic and hydrogen-bonding interactions [7,8], but can also interact strongly with metal ions through coordination, offering opportunities to tailor film structure and functionality beyond those of simple physical composites [11].

To overcome the limited functionality of neat chitosan films, the incorporation of natural plant extracts has been widely explored as a strategy for developing multifunctional active packaging materials [12,13,14,15]. *Terminalia catappa* leaves represent a valuable yet underexplored source of such bioactive compounds. The leaf extract is rich in hydrolysable tannins, gallotannins, and flavonoids [16,17], which have been associated with strong antioxidant activity through radical scavenging and inhibition of lipid oxidation [18,19]. In addition, *T. catappa* extract has shown antimicrobial activity against several foodborne microorganisms [20,21], which has been attributed to the ability of tannins and flavonoids to disrupt microbial cell membranes and interfere with essential cellular processes [16,19,22].

In parallel, zinc oxide nanoparticles (ZnO NPs) have been extensively investigated as active components in food-packaging systems [23,24]. Their reported functions are commonly interpreted within a solid-particle framework, in which nanoscale ZnO acts as an antimicrobial agent and reinforcing nanofiller [25,26,27]. However, an important physicochemical consideration is that ZnO is susceptible to acid-mediated dissolution and/or structural transformation in acidic aqueous media [28]. Because chitosan film-forming solutions are typically prepared in dilute organic acids such as acetic acid, the actual chemical state of zinc after film formation may differ substantially from that of the initial ZnO powder. This raises an important mechanistic question as to whether the final film properties arise from intact ZnO particles, zinc-containing species generated during casting, or a combination of both.

When zinc species coexist with polyphenol-rich extracts in a chitosan matrix, additional interactions may occur, including metal coordination, competitive binding, and changes in polymer network organisation [29]. Nevertheless, the combined incorporation of *T. catappa* leaf extract (TE) and ZnO into chitosan-based films has not been systematically investigated, particularly from the standpoint of zinc speciation and its structural consequences in an acidic casting system. Therefore, this study aimed to develop and characterise chitosan-based active packaging films containing TE and zinc oxide. Particular emphasis was placed on clarifying the structural state of zinc in the final films using multi-technique analyses, including X-ray diffraction (XRD), SEM–EDX, and ATR-FTIR, and on relating these findings to the resulting mechanical, barrier, antioxidant, and antimicrobial properties. It was hypothesised that TE would primarily contribute antioxidant and light-screening functions, whereas zinc-containing species derived from ZnO during film casting would modify matrix organisation and functional performance through coordination-mediated interactions within the chitosan-based network. The practical potential of the resulting films was further explored through a preliminary visual assessment using postharvest bananas.

## 2. Materials and Methods

### 2.1. Chemicals and Reagents

#### 2.1.1. Chitosan

Food-grade chitosan oligomer, derived from king crab shell (*Paralithodes camtschaticus*), was procured from Union Science (Chiang Mai, Thailand). According to the supplier’s certificate of analysis, the material had a degree of deacetylation of 92% (determined by potentiometric titration) and a viscosity of <5 cps, measured at 0.25 g in 50 mL of 0.5% acetic acid (lot no. 20211017). These supplier specifications are consistent with a low-molecular-weight chitosan grade, although an absolute molecular weight was not provided. The certificate of analysis is included in the Supplementary Materials, while the experimentally determined molecular weight is described separately in Section 2.1.3.

#### 2.1.2. Other Chemicals and Reagents

Zinc nitrate hexahydrate (Zn(NO_3_)_2_·6H_2_O) was supplied by Kemaus (Cherrybrook, Australia). Potassium bromide (KBr, FTIR grade) was obtained from Merck (Darmstadt, Germany). Potassium hydroxide (KOH) and glycerol (99.5%) were purchased from QRëC (Auckland, New Zealand). Folin–Ciocalteu’s phenol reagent and potassium persulphate were obtained from Loba (Mumbai, India). 2,2-Diphenyl-1-picrylhydrazyl (DPPH), 2,2′-azino-bis(3-ethylbenzothiazoline-6-sulfonic acid) (ABTS), Trolox, and gallic acid monohydrate (ACS grade) were purchased from Sigma-Aldrich (St. Louis, MO, USA). Deionized water was used throughout the extraction and synthesis procedures.

#### 2.1.3. Determination of Chitosan Molecular Weight

The weight-average molecular weight (Mw) of chitosan was determined by static light scattering using a Zetasizer Ultra (Malvern Panalytical, Worcestershire, UK) with ZS Xplorer software, version 3.3.0.42. Chitosan was dissolved in 0.1 M acetic acid/0.1 M NaCl and measured at 25 °C using the concentration trend mode at 173° backscatter (scattering angle ≈ 73°). The dispersant and all chitosan solutions were filtered through 0.22 μm membrane filters before analysis, and the corresponding solvent was measured as a blank for scattering correction.

Two independent concentration series were prepared: series A (0.5, 1.12, 1.75, 2.38, and 3.0 mg/mL) and series B (0.3, 0.7, 1.3, 2.0, and 3.0 mg/mL). Each concentration was analyzed in triplicate. Debye plots of KC/R versus concentration are shown in Figure S22, and the corresponding fitting parameters are summarized in Table S6. The calculations were performed using a solvent refractive index of 1.333, a refractive index increment dn/dc = 0.170 mL/g [30], and the default viscosity value for water-like media at 25 °C. Series A yielded Mw = 19.9 kDa (R^2^ = 0.87), while series B gave Mw = 23.6 kDa (R^2^ = 0.89), indicating an effective weight-average molecular weight of approximately (2.0–2.4) × 10^4^ Da for the commercial chitosan oligomer used in this study.

### 2.2. Microwave-Assisted Extraction and Drying of Terminalia catappa Leaf Extract

#### 2.2.1. Preparation of *Terminalia catappa* Leaf Powder

*Terminalia catappa* leaves were collected in Chiang Mai Province, Thailand, in January 2024. Dark red leaves were selected, washed with water, cut into small pieces, and sun-dried. The dried leaves were then ground using a blender and sieved through a 300 μm mesh. The resulting powder was packed in aluminium bags and stored in a glass desiccator at room temperature (approximately 30 °C) until use. The moisture content of the sun-dried leaf powder was determined using a moisture balance (MB120, OHAUS Corporation, Parsippany, NJ, USA) at 105 °C with an automatic switch-off criterion of 0.10%/60 s (n = 4, approximately 5 g per replicate), and was found to be 7.04 ± 0.04% (wet basis). Extraction yields reported in Section 3.1.1 are expressed on a dry-weight basis.

#### 2.2.2. Extraction

*Terminalia catappa* leaf extract was prepared by microwave-assisted extraction (MAE). Briefly, 20 g of leaf powder were extracted with 400 mL of distilled water (solid-to-solvent ratio 1:20, *w*/*v*) using a modified domestic microwave oven (R-2000W, Sharp, Shanghai, China) adapted for extraction. Extraction conditions were evaluated within the operating range of the instrument by varying microwave power (130 and 440 W) and extraction time (5, 10, 15, and 20 min). After extraction, the suspensions were filtered through Whatman No. 1 filter paper.

#### 2.2.3. Comparative Drying Methods

The filtered *T. catappa* leaf extracts were subsequently dried using two different methods to compare their effects on bioactive compound retention.

For freeze drying (FD), the extracts were pre-frozen at −20 °C for 24 h and then freeze-dried using a benchtop freeze dryer (Labconco, Kansas City, MO, USA) operated at −50 °C under high vacuum (<0.1 mbar) for 48–72 h until complete sublimation was achieved.

For vacuum drying (VD), the extracts were dried in a vacuum oven (VD23, Binder, Tuttlingen, Germany) at 45 °C under reduced pressure (<10 mbar) for 12–24 h until constant weight was reached. Vacuum drying was performed in the dark to minimise oxidative degradation.

The dried extracts were packed in aluminium bags and stored at room temperature until further analysis. Extraction yield was calculated from the dried extract mass (W_2_) relative to the initial mass of leaf powder (W_1_) using Equation (1):

(1) $$\mathrm{Yield}\;\left(\%\right)\;=\;\left(\frac{W_{2}}{W_{1}}\right)\times \;100$$

#### 2.2.4. UV–Visible Spectroscopy

UV–visible absorption spectra of *Terminalia catappa* leaf extract were recorded over the wavelength range of 250–600 nm using a UV–Vis spectrometer (V-730, JASCO, Tokyo, Japan).

#### 2.2.5. FTIR Analysis of *Terminalia catappa* Leaf Extract

Solid samples of *T. catappa* leaf extract (0.1 g) were finely ground, mixed with KBr powder, and compressed into discs using a hydraulic press. FTIR spectra were recorded using an FTIR spectrometer (FTIR-4700, JASCO, Tokyo, Japan) over the wavenumber range of 4000–400 cm^−1^ at a resolution of 4 cm^−1^ in transmittance mode.

#### 2.2.6. Total Phenolic Content (TPC)

Total phenolic content (TPC) was determined using the Folin–Ciocalteu (FC) method. Briefly, 30 μL of diluted extract (1:100) were added to a 96-well microplate, followed by 60 μL of 10% FC reagent. After incubation at room temperature for 1 min, 210 μL of 6% NaHCO_3_ solution were added. The plate was then incubated in the dark at room temperature for 90 min. Absorbance was measured at 725 nm using a multimode microplate reader (Spark, Tecan, Männedorf, Switzerland). Gallic acid was used to construct the standard curve over the concentration range of 0–200 mg/mL. TPC was expressed as mg gallic acid equivalents per gram of extract (mg GAE/g extract).

#### 2.2.7. Antioxidant Assays of *Terminalia catappa* Leaf Extract

##### DPPH Analysis

DPPH radical scavenging activity was determined according to the method of Brand-Williams et al. [31] with microplate modifications. A DPPH stock solution was prepared by dissolving 3.94 mg of DPPH in 50 mL of methanol to obtain a final concentration of 0.2 mM. The working solution was then adjusted with methanol to an absorbance of 1.00 ± 0.05 at 517 nm.

For the assay, 25 μL of extract solution at the desired concentration (prepared in methanol/water, 4:1 *v*/*v*) were mixed with 250 μL of DPPH working solution in a 96-well plate. After gentle shaking, the mixture was incubated in the dark at room temperature for 30 min. Absorbance was measured at 517 nm using a microplate reader (Spark, Tecan, Männedorf, Switzerland). Radical scavenging activity was calculated using Equation (2):

(2) $$\mathrm{DPPH}\;\mathrm{Scavenging}\;\mathrm{activity}\;\left(\%\right)\;=\;\left[\frac{A_{0}-A_{1}}{A_{0}}\right]\times 100$$

where $A_{0}$ is the absorbance of the control (DPPH solution without sample) and $A_{1}$ is the absorbance of the sample. Results were expressed as percentage radical scavenging activity and as Trolox equivalent antioxidant capacity (TEAC), reported as mg Trolox/g extract or mg Trolox/g film, depending on the sample type. TEAC values were obtained using a Trolox standard curve (0–240 mg/L in methanol, 9 concentration levels, analysed in triplicate). Sample TEAC values were calculated by interpolating the measured percentage inhibition against the corresponding Trolox calibration curve. All measurements were performed in triplicate with appropriate blank correction.

##### ABTS Analysis

ABTS•^+^ radical scavenging activity was determined according to the method of Re et al. [32] with microplate modifications. Stock solutions of 7 mM ABTS and 2.45 mM potassium persulfate were prepared separately and mixed in equal volumes. The mixture was kept in the dark at room temperature for 14–16 h to generate the ABTS•^+^ radical.

Before analysis, the ABTS•^+^ solution was diluted with phosphate buffer (pH 7.4) to obtain an absorbance of 0.70 ± 0.02 at 734 nm. Then, 10 μL of diluted extract were mixed with 200 μL of ABTS•^+^ working solution in a 96-well plate. After incubation in the dark for 6 min, absorbance was measured at 734 nm. Radical scavenging activity was calculated using Equation (3):

(3) $$\mathrm{ABTS}\;\mathrm{scavenging}\;\mathrm{activity}\left(\%\right)\;=\;[\frac{A_{0}-A_{ABTS}}{A_{0}}]\;\times \;100$$

where $A_{0}$ is the absorbance of the control (ABTS•^+^ solution without sample) and $A_{ABTS}$ is the absorbance of the sample. As in the DPPH assay, the results were expressed as percentage radical scavenging activity and TEAC, reported as mg Trolox/g extract or mg Trolox/g film, as appropriate. TEAC values were calculated using the same Trolox calibration approach described above. All determinations were performed in triplicate.

### 2.3. Synthesis of ZnO by the Hydrothermal Method

#### 2.3.1. Preparation of Hydrothermally Synthesised ZnO Powder

Zinc oxide nanostructures were synthesised by the hydrothermal method using zinc nitrate hexahydrate and potassium hydroxide as precursors, following an established procedure with minor modifications [33,34]. Briefly, Zn(NO_3_)_2_·6H_2_O (4.06 g, 0.0136 mol) and KOH (7.65 g, 0.1364 mol) were dissolved in deionized water to a final volume of 100 mL, corresponding to a Zn^2+^:OH^−^ molar ratio of 1:10, under continuous stirring. After complete dissolution, the reaction mixture was transferred into a PTFE-lined stainless-steel autoclave and heated at 150 °C for 12 h. After the reaction, the autoclave was allowed to cool naturally to room temperature. The resulting precipitate was collected by centrifugation at 5000 rpm for 10 min and washed repeatedly with deionized water until the supernatant reached pH 7. The product was then freeze-dried and stored at 30 °C until further use. A single batch of the synthesised ZnO powder was used for all subsequent film preparations in order to avoid batch-to-batch variation.

#### 2.3.2. XRD Analysis of Synthesised ZnO Powder

The crystal structure of the synthesised ZnO powder was analysed by X-ray diffraction using a MiniFlex diffractometer (Rigaku Corporation, Tokyo, Japan) with Cu-Kα radiation (λ = 1.549 Å) over a 2θ range of 20–80° at a scanning speed of 0.02° s^−1^ [35]. The instrument was operated at 40 kV and 30 mA. The average crystallite size (D) was estimated using the Scherrer equation (Equation (4)):

(4) $$D\;=\;\frac{0.89\lambda }{\beta \mathrm{Cos}\theta_{B}}$$

#### 2.3.3. Scanning Electron Microscopy (SEM)

The morphology of the synthesised ZnO was examined by scanning electron microscopy (SEM) using a JEOL JSM-IT200 instrument (JEOL, Tokyo, Japan). Prior to imaging, the samples were sputter-coated with a thin layer of gold to improve conductivity. The SEM was operated at an accelerating voltage of 20 kV under vacuum.

#### 2.3.4. FT-IR Analysis

The functional groups of the synthesised ZnO powder were analysed by FTIR spectroscopy using an FTIR-4700 spectrometer (JASCO, Tokyo, Japan). Briefly, 0.1 g of ZnO powder was mixed with potassium bromide (KBr) and compressed into a pellet. Spectra were recorded over the range 4000–400 cm^−1^ at a resolution of 4 cm^−1^.

#### 2.3.5. Thermogravimetric Analysis (TGA)

The thermal stability of the synthesised ZnO powder was evaluated by thermogravimetric analysis using a TGA/DSC 3+ instrument (Mettler Toledo, Greifensee, Switzerland). Approximately 5 mg of sample were placed in an aluminium oxide crucible and analysed from 30 to 800 °C at a heating rate of 10 °C min^−1^ under a nitrogen flow of 20 mL min^−1^.

### 2.4. Chitosan-Based Active Packaging Films Incorporating Terminalia catappa Leaf Extract and Zinc Oxide

#### 2.4.1. Preparation of the Films

Sixteen formulations of chitosan-based active packaging films were prepared by the solvent-casting method [36]. First, chitosan (1.5%, *w*/*v*) was dissolved in 1.0% (*v*/*v*) aqueous acetic acid at 60 °C under continuous stirring, followed by the addition of glycerol (0.5%, *v*/*v*) as a plasticiser [37]. The mixture was stirred using an overhead stirrer for 30 min to ensure homogeneity. Subsequently, *T. catappa* leaf extract (TE) and the synthesised ZnO powder were added to the chitosan solution to obtain final additive concentrations of 0, 0.1, 0.2, and 0.3% (*w*/*v*). The resulting films were designated as TxZy, where T and Z represent the TE concentration and the initial ZnO loading, respectively, and x and y denote concentration levels from 0 to 3 (Table S1). The mixtures were further stirred, ultrasonicated for 30 min to remove entrapped air bubbles, and then cast (30 mL) onto PTFE-coated dishes (16 cm in diameter). The films were dried in a hot-air oven at 55 °C for 12 h and stored in a desiccator until further characterisation.

#### 2.4.2. Colour Properties

The colour properties of the films were determined using a colorimeter (NH300, 3nh, Shenzhen, China) [38]. The instrument was calibrated against a white standard plate before measurement. Each film was measured at five random positions, and the average CIELAB values (L*, a*, and b*) were recorded. The total colour difference (ΔE) relative to the control film (T0Z0) was calculated using Equation (5):

(5) $$∆E\;=\;\sqrt{{\left(L^{*}-L_{0}^{*}\right)}^{2}+{\left(a^{*}-a_{0}^{*}\right)}^{2}+{\left(b^{*}-b_{0}^{*}\right)}^{2}}$$

where $L_{0}^{*},\;a_{0}^{*}$ and $b_{0}^{*}$ are the colour values of the control film, and $L^{*},\;\;a^{*}\;$ and $b^{*}$ are those of the composite film.

#### 2.4.3. Haze and Transmittance Properties

Haze and transmittance properties of the films were measured using a YH1200 haze meter, GBPI (GuangZhou Biaoji Packaging Equipment Co., Ltd., Guangzhou, China), at room temperature in accordance with ASTM D1003 and ISO 13468 standards.

#### 2.4.4. Scanning Electron Microscopy (SEM) and Energy-Dispersive X-Ray Spectroscopy (EDX)

The surface morphology of the composite films was investigated using a scanning electron microscope (Prisma E, Thermo Fisher Scientific, Hillsboro, OR, USA) operated at 15 kV. Elemental composition was determined by energy-dispersive X-ray spectroscopy (X-Act, Oxford Instruments, Wycombe, UK) coupled to the SEM. A thin coating of conductive gold was applied to the samples prior to imaging.

#### 2.4.5. XRD Analysis of Composite Films

XRD patterns of all 16 film formulations were recorded using the same diffractometer and conditions described in Section 2.3.2 (MiniFlex, Rigaku Corporation, Tokyo, Japan; Cu-Kα, 30 mA, 40 kV, 2θ = 20–80°, 0.02°/s) to assess the crystalline state of ZnO within the dried film matrix.

#### 2.4.6. Thickness and Mechanical Properties

Film thickness was measured at ten random positions on each sample using a desktop thickness gauge (GT-313-A, Gotech Testing Machines Inc., Dongguan, China). Tensile strength (TS) and percentage of elongation at break (EAB) were measured using a universal testing machine (H-1KS, Hounsfield, Redhill, UK) following the ASTM D882-02 standard [39]. Film specimens were cut into 15 × 100 mm strips and mounted into tensile grips with a 1000 N load cell. The initial gauge length and stretching speed were set at 50 mm and 20 mm/min, respectively. Tensile strength calculations were intrinsically normalized by the actual cross-sectional area (width × measured thickness) of each specific specimen, thereby accounting for any formulation-induced thickness variations.

#### 2.4.7. ATR-FTIR Analysis of Composite Films

The chemical interactions within the composite films were analyzed using the FTIR spectrometer described in Section 2.2.5. The spectra were recorded with the wavelength at 4000–400 cm^−1^.

#### 2.4.8. Moisture Content

Moisture content percentage of the composite films was determined using a moisture analyzer (MB120, OHAUS, USA). Five grams of the film were placed in the moisture analyzer pan, and the measurement was performed at 105 °C until constant weight. Given that gravimetric drying at 105 °C may co-volatilize trace residual acetic acid (boiling point ~118 °C) physically entrapped within the polymer matrix, the reported values represent total volatile content, which might slightly overestimate the absolute water mass.

#### 2.4.9. Water Vapour Permeability (WVP) and Water Vapour Transmission Rate (WVTR)

Water vapour transmission rate (WVTR) and water vapour permeability (WVP) were determined using a water vapour transmission rate test system (C303H, Labthink, Jinan, China) in accordance with ASTM F1249-20 and ISO 15106-2 [40]. Both WVTR and WVP values were obtained directly from the instrument software, which calculated WVP from the measured WVTR, film thickness, and the applied partial pressure gradient.

#### 2.4.10. Contact Angle (CA)

Surface wettability of the films was evaluated using a drop shape analyser (DSA30, Krüss, Hamburg, Germany) at room temperature. The contact angle of a sessile water droplet on the film surface was measured and expressed as the average of both sides of the droplet [41].

#### 2.4.11. Thermal Analysis of Composite Films (DSC and TGA)

Thermal stability (TGA) and phase transitions (DSC) of the films were evaluated simultaneously using the TGA/DSC analyzer described in Section 2.3.5. Analysis was performed at a heating rate of 10 °C/min under a nitrogen flow of 20 mL/min. DSC parameters were recorded from 25 to 350 °C, while TGA measurements were conducted from 30 to 800 °C.

#### 2.4.12. Disc-Diffusion Antimicrobial Assay of Composite Films

An antimicrobial activity of the films was evaluated by the disc diffusion method. Two types of microorganisms were employed: *Staphylococcus aureus* (Gram-positive) and *Escherichia coli* (Gram-negative) [42]. The nutrient broth was used to make a bacterial suspension and incubated at 37 °C for 8 h. The cultures were then adjusted to reach a turbidity of about 1.5 × 10^8^ CFU/mL, which is the same as the 0.5 McFarland standard. 100 µL of each modified bacterial suspension was then evenly distributed onto sterile Nutrient Agar (NA) plates using a sterile glass spreader. Film samples were cut into circular discs (10 mm diameter) using a cork borer and placed on the surface of inoculated agar plates. The 10 mm disc size was selected to ensure sufficient film–agar contact for evaluating antimicrobial agent diffusion from the composite films. The dishes were incubated at 37 °C for 24 h. Following incubation, the diameter of the inhibition zones (including the film) was determined using a vernier calliper. The results were reported as the mean ± standard deviation in millimetres, and each test was conducted in triplicate.

#### 2.4.13. Antioxidant Activity of Film Extracts

To obtain the film extract, 0.05 g of the film sample was immersed in 10 mL of 1% (*v*/*v*) acetic acid for 30 min at room temperature. The DPPH and ABTS radical scavenging activities of the resulting extracts were then determined strictly following the microplate protocols and equations described in Section 2.2.7. For TEAC determination, Trolox standard solutions (0, 30, 60, 90, 120, 150, 180, 210, and 240 mg/L in methanol) were subjected to both DPPH and ABTS assays under identical conditions as the film samples. The percentage radical scavenging activity was plotted against Trolox concentration, and linear regression was performed to obtain standard curves (DPPH: y = 0.4121x − 1.09, R^2^ = 0.9972; ABTS: y = 0.4458x + 2.76, R^2^ = 0.9926). The TEAC of each film sample was calculated by interpolating the percentage inhibition value onto the respective standard curve and expressing the result as mg Trolox equivalents per gram of film (mg Trolox/g film).

### 2.5. Preliminary Visual Quality Assessment of Packaged Bananas

To preliminarily assess the practical potential of the composite films for food-packaging applications, a visual quality assessment was conducted using Namwa bananas (Pisang Awak, *Musa* ABB group) following previously reported postharvest observation approaches [43,44]. Bananas free from visible disease and mechanical damage were selected from a single batch in order to minimise initial physiological variation. After washing with distilled water to remove surface impurities and drying under ambient conditions, the bananas were individually wrapped with the composite films. Unwrapped bananas from the same batch were used as the control. All samples were stored at ambient temperature (25 ± 2 °C) for 7 days. This trial was intended solely as a preliminary visual screening; therefore, the assessment was limited to photographic observation of peel colour change and browning. Quantitative postharvest parameters and post-application changes in film properties were not evaluated in this preliminary test.

### 2.6. Statistical Analysis

All statistical analyses were performed using IBM SPSS Statistics 17.0 (IBM Corp., Armonk, NY, USA). Data are presented as mean ± standard deviation (n ≥ 3). Prior to inferential analysis, data normality and homogeneity of variance were assessed using the Shapiro–Wilk test and Levene’s test, respectively. When the assumptions for parametric analysis were satisfied, differences among treatments were evaluated by one-way analysis of variance (ANOVA), followed by Tukey’s honestly significant difference (HSD) post hoc test at a significance level of *p* < 0.05. Graphs were generated using OriginPro 2021 (OriginLab, Northampton, MA, USA).

## 3. Results and Discussion

### 3.1. Microwave-Assisted Extraction of T. catappa Leaf Extract

#### 3.1.1. Temperature Profile, Extraction Yield, Total Phenolic Content, and Antioxidant Activities

**Figure 1:**
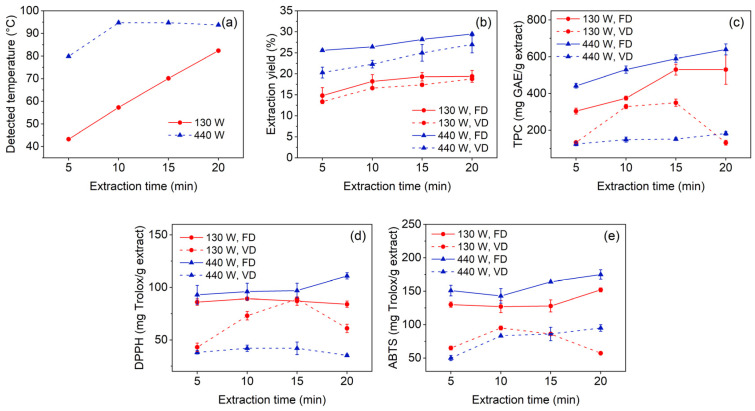
Effects of microwave power (130 and 440 W), extraction time (5, 10, 15 and 20 min), and drying method on (**a**) detected temperature, (**b**) extraction yield, (**c**) total phenolic content, (**d**) DPPH, and (**e**) ABTS radical scavenging activities of *Terminalia catappa* leaf extracts. FD: freeze drying; VD: vacuum drying.

Figure 1 summarises the effects of microwave power (130 and 440 W) and extraction time (5, 10, 15, and 20 min) on the detected temperature, extraction yield, total phenolic content (TPC), and antioxidant activities of *T. catappa* leaf extract. The detected temperature increased with both microwave power and extraction time (Figure 1a). At 130 W, the temperature rose gradually from 43 °C at 5 min to 82 °C at 20 min. In contrast, extraction at 440 W increased the temperature of the aqueous medium to approximately 80 °C within 5 min and approached 95 °C after 10 min, which is close to the boiling point of water under atmospheric conditions. Beyond this point, additional microwave energy was likely dissipated mainly through solvent evaporation rather than further bulk temperature increase.

Extraction yields ranged from 14.0 to 29.5% on a dry-weight basis across all tested conditions (Table S2 and Figure 1b), with higher yields generally obtained at longer extraction times and higher microwave power. This trend is consistent with enhanced disruption of plant tissue and improved diffusion of soluble compounds into the aqueous phase. At all time points, extraction at 440 W gave higher yields than extraction at 130 W. The highest yield was obtained at 440 W for 20 min followed by freeze drying (29.5 ± 0.2%, dry basis), whereas the corresponding vacuum-dried extract gave a slightly lower value (27.1 ± 2.1%). These results suggest that the more rapid heating achieved at higher microwave power promoted more efficient release of extractable constituents, including phenolic compounds, from the leaf matrix [45,46].

The drying method also influenced extract recovery. Under both microwave power levels, freeze-dried extracts generally gave higher yields than vacuum-dried extracts. Freeze drying is expected to better preserve thermolabile constituents because water removal occurs by sublimation under low-temperature and low-pressure conditions. In contrast, vacuum drying at 45 °C exposes the extract to a longer period of moderate thermal stress, which may contribute to partial degradation or loss of heat-sensitive compounds during the drying process [47,48].

TPC varied markedly with both microwave power and drying method (Figure 1c). Among all tested conditions, the freeze-dried extracts obtained at 440 W gave the highest TPC values, increasing from 442 ± 13 mg GAE/g extract at 5 min to 639.5 ± 28.4 mg GAE/g extract at 20 min (Table S3). The corresponding freeze-dried extracts obtained at 130 W followed a similar increasing trend, reaching 530 ± 80 mg GAE/g extract at 20 min. In contrast, vacuum drying resulted in substantially lower TPC values. At 130 W, the vacuum-dried extract reached 350 ± 20 mg GAE/g extract at 15 min and then decreased to 132 ± 13 mg GAE/g extract at 20 min. Likewise, the 440 W vacuum-dried series did not exceed 183 ± 10 mg GAE/g extract under any of the tested conditions. These results suggest that freeze drying preserved phenolic constituents more effectively than vacuum drying, whereas prolonged thermal exposure during vacuum drying may have contributed to partial degradation of thermolabile compounds [49].

The DPPH radical scavenging activity generally followed the same trend as TPC (Figure 1d). The highest activity was observed for the freeze-dried extract obtained at 440 W for 20 min, with a value of 183 ± 10 mg Trolox/g extract. For the freeze-dried extracts obtained at 130 W, the DPPH values remained within a relatively narrow range (84 ± 3 to 86 ± 3 mg Trolox/g extract) across the tested extraction times. In the vacuum-dried series at 130 W, DPPH activity increased to 89.0 ± 0.8 mg Trolox/g extract at 15 min before decreasing to 61 ± 4 mg Trolox/g extract at 20 min, in parallel with the corresponding reduction in TPC. The vacuum-dried extracts obtained at 440 W also showed comparatively low DPPH values across all time points, consistent with their lower phenolic content [50].

A similar trend was observed for ABTS radical scavenging activity (Figure 1e), although the absolute values were consistently higher than those obtained from the DPPH assay. The freeze-dried extracts obtained at 440 W again gave the highest values, increasing from 151 ± 8 mg Trolox/g extract at 5 min to 175 ± 7 mg Trolox/g extract at 20 min, whereas both vacuum-dried series remained lower. The higher ABTS values may be attributed to differences in the reaction mechanisms of the two assays. The DPPH assay primarily reflects hydrogen atom transfer from phenolic hydroxyl groups, whereas the ABTS assay can involve both hydrogen atom transfer and single-electron transfer pathways [51]. Because *T. catappa* leaves contain hydrolysable tannins and related phenolic compounds with multiple hydroxyl functionalities, higher antioxidant values in the ABTS assay than in the DPPH assay are reasonable [52,53]. Overall, the results indicate that extraction at 440 W followed by freeze drying was the most favourable condition within the tested range for recovering phenolic constituents and preserving antioxidant activity.

#### 3.1.2. UV–Vis Spectroscopy

The UV–Vis absorption spectra (250–600 nm) of the *T. catappa* leaf extracts are shown in Figure S1. All spectra exhibited two characteristic absorption features: a major band at 259–261 nm and a weaker shoulder near 370 nm.

The band at 259–261 nm can be attributed to π → π* transitions associated with aromatic structures present in phenolic acids, flavonoids, and hydrolysable tannins, which are among the principal phytochemical classes reported in *T. catappa* leaves [54]. The shoulder near 370 nm is consistent with n → π* transitions associated with carbonyl-containing and conjugated phenolic structures, such as flavonol derivatives and ellagic acid-related compounds [55,56].

For all extraction and drying conditions, the absorbance intensity at both regions increased with extraction time, in agreement with the increasing TPC values described in Section 3.1.1. Accordingly, the freeze-dried extract obtained at 440 W for 20 min showed the highest overall absorbance, consistent with its higher phenolic content and antioxidant activity. Importantly, the general spectral profile remained similar across all tested conditions, whereas the principal variation was in band intensity. This suggests that the extraction and drying conditions mainly affected the amount of recovered phenolic constituents rather than causing major qualitative changes in the extract composition. In addition, the spectra obtained at 440 W (Figure S1b,d) showed a more pronounced shoulder near 370 nm than those obtained at 130 W (Figure S1a,c), which may indicate more efficient recovery of conjugated flavonol-type compounds at the higher microwave power.

#### 3.1.3. FTIR Analysis

Figure S2 shows the FTIR spectra (4000–400 cm^−1^) of the *T. catappa* leaf extracts prepared at 130 and 440 W and dried either by freeze drying (Figure S2a,b) or vacuum drying (Figure S2c,d). The overall spectral profiles were similar across all extraction and drying conditions, indicating that the principal functional groups of the extracted compounds were retained under the tested conditions.

A broad band in the region 3421–3743 cm^−1^ was assigned to O–H stretching vibrations associated with phenolic hydroxyl groups and residual bound moisture. A weaker band at 2927–2929 cm^−1^ corresponded to aliphatic C–H stretching. The absorption band at 1716–1717 cm^−1^ is consistent with ester C=O stretching, which has been associated with hydrolysable tannins and gallic acid derivatives reported in *T. catappa* leaves [52,54]. Additional bands at 1613 cm^−1^ and 1443–1444 cm^−1^ were attributed to aromatic ring vibrations and C–H bending, respectively. In the fingerprint region, the bands at 1348–1357 cm^−1^ and 1213–1220 cm^−1^ were assigned to phenolic C–O stretching and ester-related C–O–C vibrations, whereas the band at 1057–1059 cm^−1^ corresponded to C–O stretching of alcohol groups. The lower-wavenumber bands at 871–876 and 757–758 cm^−1^ were assigned to aromatic C–H out-of-plane bending [55].

The relative intensities of several of these bands, particularly those associated with O–H and ester C=O functionalities, tended to increase with longer extraction time, especially at 440 W. This trend is consistent with the greater recovery of phenolic constituents indicated by the TPC and UV–Vis results. Taken together, the FTIR data support the successful extraction of phenolic-rich constituents from *T. catappa* leaves without obvious alteration of the principal functional groups under the tested conditions. It should be noted, however, that the UV–Vis and FTIR analyses in this study were used as qualitative fingerprinting tools to indicate the presence of broad phytochemical classes. Detailed identification and quantification of individual compounds would require more specific analytical techniques, such as HPLC-DAD or LC-MS/MS, and were beyond the scope of the present work.

#### 3.1.4. Selection of Preferred Extraction Conditions

Based on the combined results for extraction yield, TPC, antioxidant activity, UV–Vis absorption characteristics, and FTIR profiles, the preferred extraction condition within the tested range was 440 W for 20 min followed by freeze drying. Under this condition, the extract showed the highest yield (29.5 ± 0.2%, dry basis), the highest TPC (639.5 ± 28.4 mg GAE/g extract), and the highest antioxidant activities in both the DPPH and ABTS assays (183 ± 10 and 175 ± 7 mg Trolox/g extract, respectively).

These values compare favourably with those commonly reported for conventional extraction approaches applied to *T. catappa* leaves and related plant matrices, particularly when considering the relatively short extraction time used in the present study. Nevertheless, direct comparison among studies should be made with caution because the reported values may be influenced by differences in plant origin, maturity, sample preparation, particle size, solvent system, and analytical protocol.

It should also be noted that the present extraction study was conducted within a defined experimental range involving two microwave power levels and four extraction times. Although the selected condition performed best within this range, broader optimisation incorporating additional variables, such as solvent composition, solid-to-solvent ratio, and particle size, may identify further improvements. On this basis, the freeze-dried extract obtained at 440 W for 20 min was selected for subsequent preparation of the chitosan-based active packaging films described in Section 3.3.

### 3.2. Synthesis and Characterization of ZnO Nanostructures

#### 3.2.1. UV–Vis Spectroscopy and Optical Band Gap

**Figure 2:**
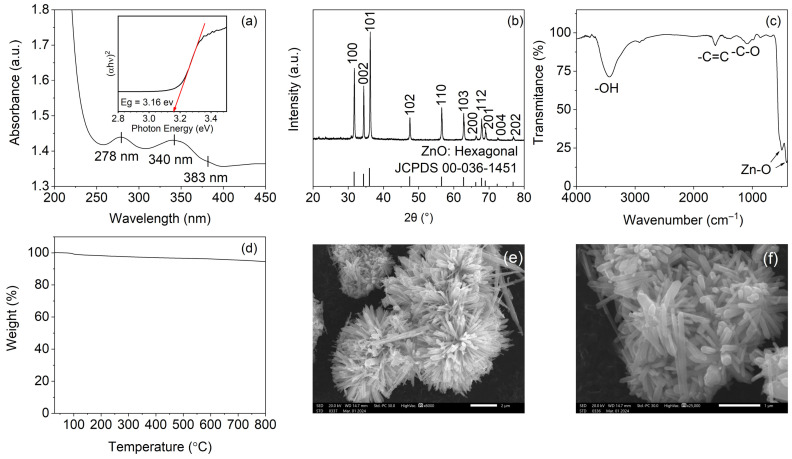
Characterization of hydrothermally synthesised ZnO nanostructures: (**a**) UV–Vis absorption spectrum with Tauc plot (inset), in which the red arrow indicates the linear extrapolation used to estimate the optical band gap of 3.16 eV, (**b**) XRD pattern indexed to hexagonal wurtzite structure (JCPDS 00-036-1451), (**c**) FTIR spectrum (4000–400 cm^−1^), (**d**) TGA thermogram recorded from 30 to 800 °C under nitrogen atmosphere, (**e**) SEM micrograph at ×800, and (**f**) SEM micrograph at ×25,000 showing flower-like hierarchical morphology composed of hexagonal nanorods.

Figure 2a shows the UV–Vis absorption spectrum of the ZnO powder prepared by the hydrothermal method. Three absorption features were observed at approximately 278, 340, and 383 nm. The band at 278 nm may be associated with defect-related transitions involving oxygen vacancies or zinc interstitials within the ZnO lattice [56]. A shoulder near 340 nm was consistent with bound-exciton absorption, whereas the absorption edge at 383 nm corresponded to the near-band-edge transition of ZnO arising from valence-to-conduction-band excitation [57,58].

The optical band gap ($E_{g}$) was estimated from the Tauc plot $(\alpha h\nu {)}^{2}$ versus hν, assuming a direct allowed transition (inset, Figure 2a). Extrapolation of the linear region to the abscissa gave a band gap of 3.16 eV, which is slightly lower than the commonly cited bulk ZnO value of 3.37 eV. Such a shift may be related to defect states, surface effects, and morphology-dependent structural features in hydrothermally synthesised ZnO [59]. The obtained value falls within the range reported for ZnO with different nanoscale morphologies [60,61], supporting the successful formation of nanostructured ZnO.

#### 3.2.2. X-Ray Diffraction

The XRD pattern of the synthesised ZnO is presented in Figure 2b. All diffraction peaks could be indexed to the hexagonal wurtzite structure of ZnO (JCPDS 00-036-1451, space group P6_3_mc). Reflections at 2θ ≈ 31.8°, 34.4°, 36.3°, 47.5°, 56.6°, 62.9°, 66.4°, 67.9°, 69.1°, 72.6°, and 76.9° were assigned to the (100), (002), (101), (102), (110), (103), (200), (112), (201), (004), and (202) planes, respectively [58]. The strong and well-defined diffraction peaks indicate that the product was highly crystalline. No peaks attributable to zinc hydroxide or other secondary phases were detected, suggesting that the synthesised powder was phase-pure within the detection limits of the technique.

Crystallite size was estimated using the Scherrer equation applied to the three strongest reflections, namely (100), (002), and (101), yielding values of 82.97, 92.78, and 69.96 nm, respectively. These coherent diffraction domain sizes are smaller than the rod diameters observed by SEM (Section 3.2.5), suggesting that individual ZnO rods may contain more than one crystalline domain rather than behaving as single crystals.

#### 3.2.3. FTIR Spectroscopy

Figure 2c shows the FTIR spectrum of the synthesised ZnO powder. The most prominent feature was a strong broad absorption below 600 cm^−1^, which is characteristic of Zn–O stretching vibrations in ZnO [59]. A broad band near 3421 cm^−1^ was attributed to O–H stretching of adsorbed water and surface hydroxyl groups. Weak bands at approximately 1613 and 1059 cm^−1^ may be associated with minor organic residues or surface-associated species originating from the aqueous synthesis route [62]. The low intensity of these bands, together with the absence of obvious nitrate- or carbonate-related absorptions, suggests that the washing procedure substantially removed precursor-derived residues from the final product.

#### 3.2.4. Thermogravimetric Analysis

The TGA curve recorded from 30 to 800 °C under nitrogen (Figure 2d) showed only a small overall mass loss of approximately 5%, leaving about 95% residual mass at the end of the scan. An initial 2–3% loss below 200 °C was attributed to the removal of physically adsorbed water and surface hydroxyl species. A further minor decrease between 200 and 400 °C may be associated with the decomposition of trace residual organics and dehydroxylation of chemisorbed species [63]. Above 400 °C, the mass remained essentially constant. These results indicate that the synthesised ZnO powder possessed high thermal stability and low residual impurity content.

#### 3.2.5. Scanning Electron Microscopy

SEM micrographs obtained at ×8000 and ×25,000 magnification (Figure 2e,f) showed that the synthesised product exhibited a flower-like morphology composed of multiple nanorods radiating from common nucleation centres to form spherical aggregates approximately 3–5 μm in diameter. At higher magnification, the individual rods displayed hexagonal cross-sections with widths of approximately 100–200 nm and lengths of about 500 nm to 1 μm. This morphology is consistent with preferential growth along the c-axis of the wurtzite ZnO structure under alkaline hydrothermal conditions [57,64,65,66].

These observations confirm that the hydrothermal method produced well-defined ZnO with hierarchical rod-based morphology prior to incorporation into the film-forming system. The behaviour of this precursor after dispersion in the acidic chitosan solution is discussed separately in Section 3.3.

#### 3.2.6. Summary of ZnO Characterization

Taken together, the UV–Vis, XRD, FTIR, TGA, and SEM results confirmed the successful synthesis of highly crystalline ZnO with a hexagonal wurtzite structure by the hydrothermal method. The as-synthesised material exhibited a flower-like hierarchical morphology composed of rod-shaped substructures with widths of approximately 100–200 nm, an optical band gap of 3.16 eV, and high thermal stability up to 800 °C. These results indicate that the hydrothermal procedure produced a structurally well-defined ZnO precursor suitable for subsequent incorporation into the chitosan-based film-forming system.

Dynamic light scattering (DLS) was not performed because the hierarchical and aggregated morphology of the ZnO particles would not provide a physically meaningful hydrodynamic diameter. Instead, particle dimensions were evaluated from SEM observations and crystallite size was estimated from XRD peak broadening. The behaviour of this ZnO precursor after addition to the acidic chitosan casting medium is discussed separately in Section 3.3.

### 3.3. Characterization of Chitosan-Based Active Packaging Films

#### 3.3.1. Structural and Morphological Characterization of Films: XRD, FTIR, SEM, and EDX

##### X-Ray Diffraction (XRD) Analysis

**Figure 3:**
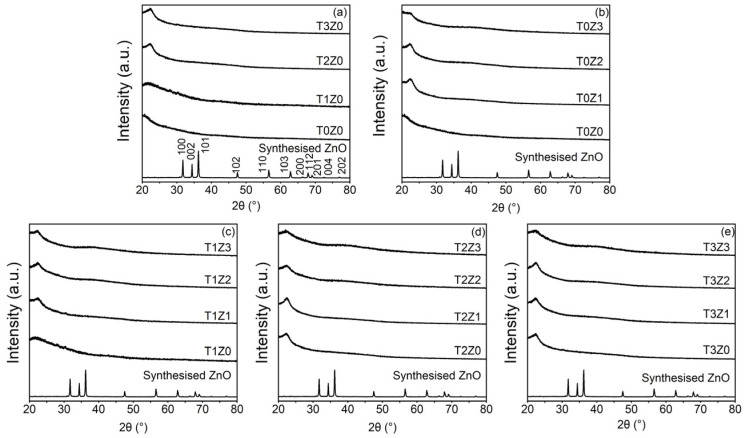
X-ray diffraction (XRD) patterns of chitosan-based composite films incorporated with varying concentrations of TE and ZnO: (**a**) films with varying TE content at 0% ZnO (T0Z0–T3Z0); (**b**) films with varying ZnO content at 0% TE (T0Z0–T0Z3); (**c**) films with varying ZnO content at 0.1% TE (T1Z0–T1Z3); (**d**) films with varying ZnO content at 0.2% TE (T2Z0–T2Z3); and (**e**) films with varying ZnO content at 0.3% TE (T3Z0–T3Z3).

The X-ray diffraction patterns of the composite films are shown in Figure 3. A notable observation is that the characteristic diffraction peaks of crystalline wurtzite ZnO, typically observed at 2θ values near 31.8°, 34.4°, and 36.3°, were no longer detectable in any of the film formulations, irrespective of the initial ZnO loading. This result indicates that the original crystalline ZnO phase was not retained after film formation. Given that the films were prepared in 1.0% (*v*/*v*) aqueous acetic acid, the XRD results are consistent with acid-mediated dissolution and/or structural transformation of ZnO during the casting process [28]. The reaction shown in Equation (6) is presented as a plausible dissolution pathway under the acidic conditions employed:

(6) $${ZnO}_{\left(s\right)}+{2CH}_{3}{COOH}_{\left(aq\right)}\to {Zn}_{\left(aq\right)}^{2+}+{2CH}_{3}{COO}_{\left(aq\right)}^{-}+H_{2}O_{\left(l\right)}$$

In addition to the disappearance of detectable ZnO diffraction peaks, the broad diffraction halo centred at approximately 2θ ≈ 20°, characteristic of the semi-crystalline chitosan matrix, became more intense and somewhat more defined with increasing zinc loading. This behaviour suggests an increase in short-range structural order or tighter chain packing within the polymer matrix rather than the persistence of ZnO as a dispersed crystalline nanofiller. A possible explanation is that zinc-containing species generated during film preparation interacted with the amino and hydroxyl groups of chitosan, thereby restricting chain mobility and promoting a more ordered arrangement of the oligomeric chitosan chains. A similar, although less pronounced, change was also observed in films containing TE alone, which may reflect additional intermolecular interactions, such as hydrogen bonding, between extract-derived polyphenols and the chitosan matrix. In the films containing both TE and zinc, these effects appeared to coexist, leading to a more structured matrix under some formulations.

##### Fourier-Transform Infrared (FTIR) Spectroscopy

**Figure 4:**
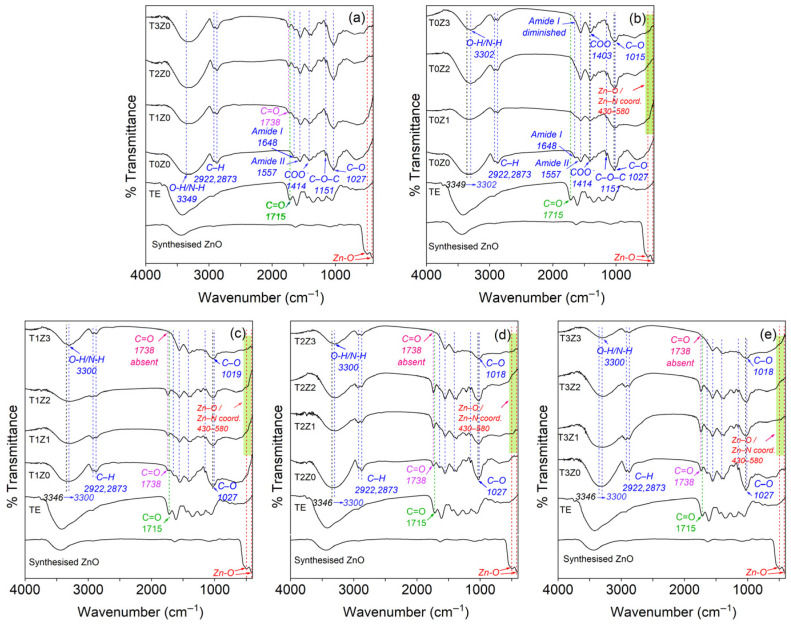
Fourier-transform infrared (FTIR) spectra of chitosan-based composite films incorporated with varying concentrations of TE and ZnO: (**a**) films with varying TE content at 0% ZnO (T0Z0–T3Z0); (**b**) films with varying ZnO content at 0% TE (T0Z0–T0Z3); (**c**) films with varying ZnO content at 0.1% TE (T1Z0–T1Z3); (**d**) films with varying ZnO content at 0.2% TE (T2Z0–T2Z3); and (**e**) films with varying ZnO content at 0.3% TE (T3Z0–T3Z3). The green shaded area highlights the low-wavenumber region (430–580 cm^−1^) associated with Zn–O and/or Zn–N coordination.

The structural changes suggested by the XRD analysis were further supported by the FTIR spectra of the films (Figure 4). The control film (T0Z0) showed the characteristic broad O–H/N–H stretching band in the 3200–3500 cm^−1^ region, together with the residual Amide I band near 1650 cm^−1^ and the Amide II band near 1550 cm^−1^, which are typical of partially deacetylated chitosan. In films containing higher zinc loadings, particularly T1Z3, T2Z3, and T3Z3, the Amide I band became markedly weaker and, in some cases, less distinguishable from the neighbouring Amide II region. This behaviour suggests a substantial change in the local bonding environment of carbonyl- and amine-containing groups, consistent with interactions between zinc-containing species and the chitosan matrix [11].

In addition, the sharp Zn–O lattice vibration typically associated with crystalline wurtzite ZnO below 550 cm^−1^ was not observed in the film spectra, in agreement with the XRD results showing no detectable crystalline ZnO phase in the dried films. Instead, a broader absorption region appeared at approximately 430–580 cm^−1^ in the zinc-containing formulations. Although band assignment in this region should be made with caution, this feature is consistent with zinc-associated coordination environments formed after film casting. In films containing both TE and zinc, the high-wavenumber O–H/N–H stretching region also became broader than in the zinc-only films, suggesting the coexistence of multiple intermolecular interactions, including hydrogen bonding and zinc-mediated coordination. It should also be noted that residual acetic acid and protonated chitosan species may contribute to parts of the carbonyl and amide regions; therefore, the FTIR interpretation is considered in conjunction with the XRD and EDX results rather than as a stand-alone proof of speciation.

##### Scanning Electron Microscopy (SEM) and Energy-Dispersive X-Ray Spectroscopy (EDX)

**Figure 5:**
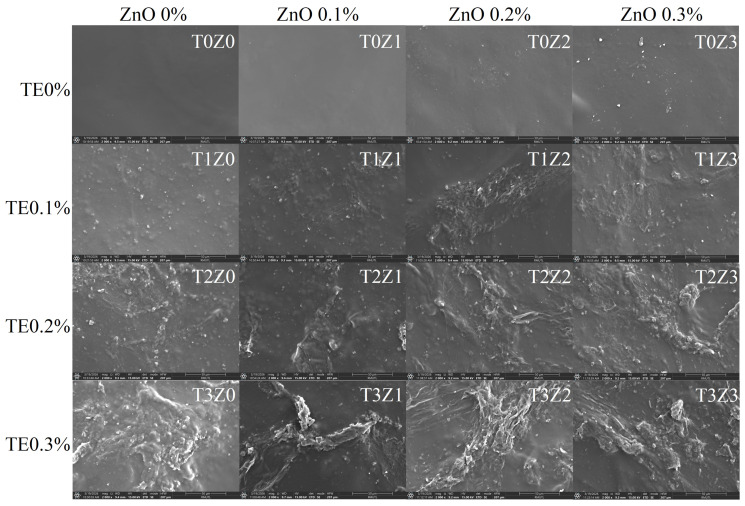
Surface morphology of chitosan-based composite films incorporated with varying concentrations of TE and ZnO observed via scanning electron microscopy (SEM) at ×2000 magnification.

The structural changes inferred from the XRD and FTIR analyses were also reflected in the surface morphology observed by SEM (Figure 5). The control chitosan film (T0Z0) exhibited a relatively smooth and homogeneous surface, which is typical of solution-cast chitosan films. The addition of TE alone (T1Z0–T3Z0) did not markedly disrupt this surface uniformity, suggesting that the extract was incorporated without obvious microscale phase separation under the conditions used.

In contrast, films containing zinc showed more heterogeneous surface features, particularly at higher nominal ZnO loadings (0.2–0.3% *w*/*v*). Because XRD showed no detectable crystalline ZnO phase in the dried films, these dispersed surface deposits are unlikely to represent intact ZnO particles. Instead, they may reflect zinc-associated domains formed during film casting and drying, possibly involving interactions among zinc-containing species, chitosan functional groups, and TE-derived polyphenols. This effect appeared to be more pronounced in formulations containing both high TE and high zinc loadings, such as T3Z3, where larger and more irregular surface features were observed. A plausible explanation is that competitive interactions between zinc-containing species and polyphenol-rich extract components promoted local heterogeneity or phase-separated domains during solvent evaporation.

The SEM observations were complemented by EDX analysis (Table 1 and Table S4, Figures S3–S18), which confirmed that zinc remained present in the dried films in a dose-dependent manner, reaching up to 16.26 wt% and 3.95 at% in formulation T1Z3. These results indicate that although the original crystalline ZnO phase was no longer detectable by XRD, zinc was retained within the film matrix after casting. Taken together, the XRD, FTIR, SEM, and EDX results support the interpretation that zinc underwent acid-mediated dissolution and/or structural transformation during film preparation, yielding zinc-containing species that influenced the final microstructure of the films. This structural reorganisation likely contributed to the mechanical, optical, and barrier properties discussed in the following sections.

#### 3.3.2. Visual Appearance and Optical Properties

**Figure 6:**
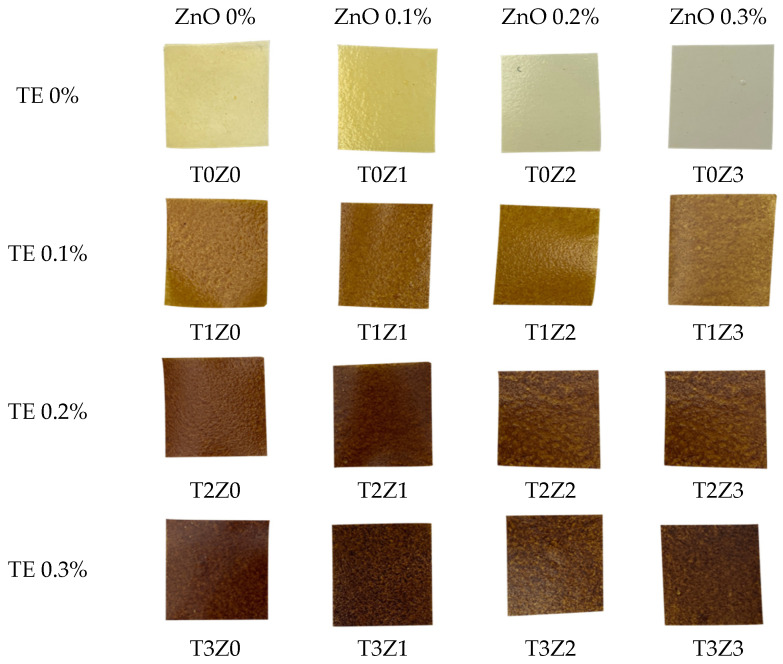
Visual appearance of chitosan-based films (2.5 × 2.5 cm) incorporated with varying concentrations of *T. catappa* leaf extract (TE: 0–0.3% *w*/*v*) and ZnO (0–0.3% *w*/*v*). Rows represent TE concentrations (0, 0.1, 0.2, 0.3% *w*/*v*); columns represent ZnO concentrations (0, 0.1, 0.2, 0.3% *w*/*v*).

The visual appearance of the films (Figure 6) and their optical properties, including colour parameters (L*, a*, b*, ΔE*), haze, and light transmittance (Table 2), provided additional macroscopic information on the effects of TE and zinc incorporation on the chitosan matrix.

In the T0 series, the control film (T0Z0) showed a pale yellow appearance, with a relatively high b* value (26.0 ± 2.0), which is consistent with the slight yellowish tone commonly observed in chitosan-based films. As the initial ZnO loading increased, the films became less yellow and visually lighter, with the b* value decreasing to 14.3 ± 0.3 in T0Z3. This change may be related to interactions between zinc-containing species and chitosan functional groups during film formation, which could alter the local chemical environment of the matrix and thereby influence film colour development. In addition, the absence of detectable crystalline ZnO in the dried films suggests that the optical behaviour of the zinc-containing films cannot be interpreted on the basis of retained particulate ZnO alone.

A corresponding improvement in visual clarity was also observed in the zinc-containing films. The haze value of the control film (T0Z0) was 54.7 ± 1.8%, whereas that of T0Z3 decreased to 29.0 ± 2.0%, while light transmittance remained above 88%. This reduction in haze is consistent with a film structure containing fewer effective light-scattering centres than would be expected from a system containing retained crystalline inorganic particles. A plausible explanation is that, under the acidic casting conditions used here, zinc-containing species became more uniformly distributed within the matrix, contributing to a more optically homogeneous film.

By contrast, the incorporation of *Terminalia catappa* extract (TE) had a pronounced effect on film appearance. Increasing TE content reduced lightness (L*) and increased redness (a*) and total colour difference (ΔE*), resulting in progressively darker films. This trend was accompanied by lower transmittance, which decreased to 21.5 ± 1.5% in T3Z3. Such behaviour is reasonably attributed to the presence of polyphenolic and flavonoid constituents in the extract, which contain chromophoric structures capable of absorbing visible light [67]. In formulations containing both high TE and high zinc levels, the films also showed greater surface heterogeneity in SEM, which may have contributed further to light scattering and reduced transparency.

From a practical perspective, this decrease in transparency may limit the suitability of high-TE formulations for applications in which direct product visibility is important. On the other hand, the darker and more opaque films may be advantageous for products that benefit from reduced light exposure. Accordingly, TE-rich films may be more suitable for light-sensitive foods or for use as inner or secondary packaging layers, whereas films with lower TE content retain better transparency for applications where visual inspection of the packaged product is desirable.

#### 3.3.3. Mechanical Properties

The mechanical properties of the active packaging films, including thickness, tensile strength (TS), and elongation at break (EAB), are summarised in Table 2. Film thickness differed among formulations, with the control film (T0Z0) showing the lowest value (0.072 ± 0.013 mm). In general, the incorporation of TE and zinc increased film thickness, with the maximum value observed for T3Z1 (0.136 ± 0.011 mm). This increase can reasonably be attributed to the greater total solids content introduced by the extract and zinc-containing components. It should be noted that TS was calculated using the actual cross-sectional area of each specimen (width × measured thickness), so the reported TS values inherently account for formulation-dependent thickness variation.

The control chitosan film (T0Z0) exhibited a TS of 6.0 ± 0.5 MPa, which is relatively low compared with values commonly reported for films prepared from higher-molecular-weight chitosan. This behaviour is consistent with the use of a low-viscosity, oligomer-grade chitosan in the present study, for which lower chain entanglement would be expected [68,69]. The addition of zinc led to a marked increase in TS, reaching 36.0 ± 4.0 MPa in T0Z3. In light of the XRD and FTIR results discussed in Section 3.3.1, this improvement is unlikely to arise from the reinforcement effect of retained crystalline ZnO particles. Rather, it is more plausibly associated with interactions between zinc-containing species and the amino and hydroxyl groups of chitosan, which may have restricted chain mobility and promoted a more ordered and mechanically resistant matrix [70].

A different behaviour was observed when high levels of both zinc and *Terminalia catappa* extract were present. Although T0Z3 showed the highest TS, the value decreased to 27.0 ± 4.0 MPa in T3Z3 despite the same initial ZnO loading. This result suggests that the effect of zinc on mechanical strength depended on the surrounding matrix composition. A plausible explanation is that, at higher TE content, extract-derived polyphenols competed with chitosan for interaction with zinc-containing species, thereby reducing the extent to which those species contributed to matrix reinforcement. The greater surface heterogeneity observed by SEM in T3Z3 is consistent with the formation of less homogeneous structural domains, which may have acted as weak points during tensile loading.

The EAB data further highlight the balance between strengthening and loss of flexibility. Formulations containing low-to-moderate levels of both additives, such as T1Z1, showed the highest flexibility, with an EAB of 37.0 ± 4.0%. At these levels, TE may have contributed a mild plasticising effect by increasing intermolecular spacing within the matrix [71], while the zinc-associated interactions were still moderate. In contrast, T3Z3 showed the lowest EAB (7.0 ± 2.0%), indicating a much more brittle response. This reduction in flexibility may be related to stronger restriction of chain movement and increased structural heterogeneity at high additive loadings.

From a practical standpoint, the results indicate a trade-off between strength and flexibility. Although T0Z3 provided the highest tensile strength, its lower extensibility may limit its usefulness in applications requiring film conformability. By comparison, intermediate formulations such as T1Z2, which combined a TS of 29.4 ± 1.2 MPa with an EAB of 32.0 ± 3.0%, showed a more balanced mechanical profile. Such formulations may therefore be more appropriate for flexible active packaging applications in which both handling resistance and adaptability to irregular product surfaces are important.

#### 3.3.4. Thermal Properties of Composite Films (DSC and TGA)

Table 3 and Figure S19 summarise the thermal transition temperatures, including glass transition temperature (Tg) and melting temperature (Tm), together with the degradation characteristics of the films, expressed as total weight loss and residual mass at 800 °C. Overall, the thermal behaviour of the films was influenced by the incorporation of TE and zinc-containing species, which altered the organisation and mobility of the chitosan-based matrix.

The control chitosan film (T0Z0) showed a Tg of 74.5 °C. The addition of TE alone (T1Z0–T3Z0) produced only a modest increase in Tg, ranging from 75.5 to 78.2 °C, suggesting that the extract contributed intermolecular interactions without markedly restricting the segmental mobility of the chitosan oligomer chains. By contrast, zinc-containing formulations showed a more pronounced increase in Tg, reaching 103.3 °C in T0Z3. In view of the XRD and FTIR results discussed in Section 3.3.1, this behaviour is consistent with stronger interactions between zinc-containing species and the chitosan matrix, which may have reduced chain mobility and increased resistance to the glass-to-rubber transition [72].

The highest Tg value was observed for T3Z3 (122.9 °C), representing an increase of approximately 48 °C relative to the control. This further increase suggests that the simultaneous presence of high TE and zinc contents led to a more constrained matrix structure. A plausible explanation is that multiple interactions, including zinc-mediated coordination and polyphenol-related intermolecular associations, contributed to the formation of a more rigid network and reduced molecular mobility in the amorphous regions of the film.

The melting temperature (Tm) varied over a narrower range, from 146.6 °C in T0Z0 to 166.4 °C in T3Z3. This comparatively modest shift suggests that the additives had a stronger influence on the amorphous regions of the films, which primarily govern Tg, than on the crystalline domains responsible for melting behaviour. This interpretation is also consistent with the XRD observations, which suggested changes in short-range order and matrix packing rather than the emergence of a new crystalline phase.

The TGA results further supported the enhanced thermal stability of the additive-containing films. Total weight loss generally decreased with the incorporation of TE and zinc, while the residual mass at 800 °C increased from 17.7% in T0Z0 to 31.2% in T3Z3. The greater residual mass may reflect the formation of thermally more stable structures and a greater tendency for carbonaceous residue formation during heating [73]. It should be noted that the TGA measurements were performed at a single heating rate (10 °C min^−1^), which does not permit kinetic analysis of degradation behaviour. Accordingly, multi-rate TGA combined with kinetic modelling would be useful in future work to provide a more rigorous evaluation of the degradation mechanisms of these ternary film systems.

#### 3.3.5. Moisture Content, Water Vapour Barrier (WVTR, WVP), and Contact Angle

The interaction of the films with water was evaluated in terms of moisture content (MC), water vapour transmission rate (WVTR), water vapour permeability (WVP), and surface contact angle (Table 4). These parameters provide further insight into how TE and zinc-containing species influenced the organisation and interfacial behaviour of the chitosan-based matrix.

The control chitosan film (T0Z0) showed a relatively high MC value (19.81 ± 0.60%), which is consistent with the hydrophilic nature of chitosan and the abundance of accessible amino and hydroxyl groups in the matrix [74]. The incorporation of zinc led to a progressive reduction in MC, reaching approximately 12% at the highest zinc loadings (e.g., T0Z3 and T3Z3). This decrease may be attributed to interactions between zinc-containing species and hydrophilic functional groups in the chitosan network, which could reduce the number of sites available for water association [75]. It should also be noted that gravimetric drying at 105 °C may include the loss of trace volatile components, such as residual acetic acid, in addition to water. Therefore, the reported MC values may slightly overestimate the absolute water content, although the comparative trend among formulations remains informative.

The water vapour barrier properties showed a similar tendency. WVTR decreased from 2.16 ± 0.07 g/(m^2^·h) in the control film to 1.72 ± 0.25 g/(m^2^·h) in T3Z3. Likewise, WVP decreased from 1.414 ± 0.033 × 10^−8^ g·cm/(cm^2^·day·Pa) in T0Z0 to 1.125 ± 0.122 × 10^−8^ g·cm/(cm^2^·day·Pa) in T0Z3. In the zinc-containing films, this improvement is consistent with a matrix that became more ordered and less permeable to water vapour, as suggested by the XRD results [75]. In films containing both TE and zinc, the barrier improvement may additionally reflect increased diffusion tortuosity arising from greater structural heterogeneity within the matrix [76]. Thus, both matrix densification and the presence of less homogeneous domains may have contributed to the reduction in water vapour transport.

Surface wettability, assessed by water contact angle, showed a related but more nuanced behaviour. The control film (T0Z0) exhibited a contact angle of 92.3 ± 9.3°. The addition of TE alone reduced the contact angle to 70.5 ± 8.0° in T3Z0, which is consistent with the introduction of polar phenolic constituents at or near the film surface [77]. In contrast, the contact angle increased to 88.3 ± 6.5° in T3Z3. This partial recovery suggests that the surface polarity of the TE-containing films was altered in the presence of zinc. A plausible explanation is that interactions between zinc-containing species and polyphenolic hydroxyl groups reduced the availability of unbound polar groups at the film surface, thereby decreasing surface wettability relative to the TE-only formulation. However, given the relatively large standard deviations, the contact-angle data should be interpreted cautiously and viewed as supportive rather than definitive evidence of surface chemical changes.

#### 3.3.6. Antioxidant Activity of Composite Films

**Figure 7:**
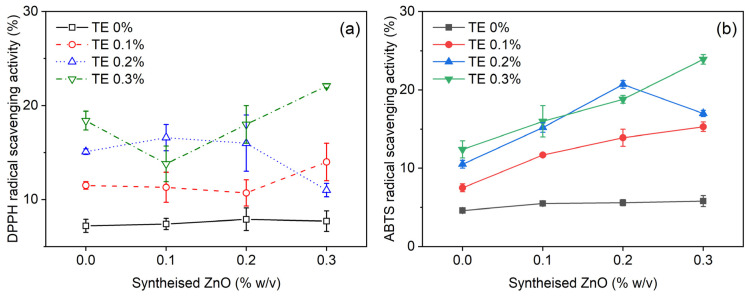
Radical scavenging activity of chitosan-based films: (**a**) DPPH and (**b**) ABTS assays. Films were prepared with TE (0, 0.1, 0.2, 0.3% *w*/*v*) and ZnO (0, 0.1, 0.2, 0.3% *w*/*v*). Values are expressed as mean ± SD (*n* = 3).

The radical scavenging activity of the films was evaluated using the DPPH and ABTS assays and expressed as Trolox equivalent antioxidant capacity (TEAC) (Table S5 and Figure 7). As expected, the control chitosan film (T0Z0) and the zinc-only formulations (T0Z1–T0Z3) showed relatively low antioxidant activity, indicating that zinc incorporation alone did not substantially contribute to TEAC under the assay conditions used. In contrast, the incorporation of *Terminalia catappa* extract (TE) clearly enhanced the antioxidant activity of the films, confirming that the extract was the principal source of radical scavenging functionality.

In the TE-only formulations, increasing TE content from T1Z0 to T3Z0 generally increased TEAC values, which is consistent with the higher loading of polyphenolic and flavonoid constituents capable of donating hydrogen atoms or electrons to neutralise free radicals [78,79]. However, the behaviour of the films containing both TE and zinc was more complex and did not follow a simple monotonic decrease with increasing zinc content. Instead, the antioxidant response depended on the specific formulation, and in several cases zinc-containing films showed TEAC values comparable to or higher than those of the corresponding TE-only films. Notably, T3Z3 exhibited the highest TEAC values in both the DPPH and ABTS assays, indicating that the presence of zinc did not necessarily suppress the measurable antioxidant activity of TE in the film matrix.

This behaviour suggests that the role of zinc in the antioxidant response is indirect and formulation-dependent. In addition to possible interactions between zinc-containing species and TE-derived polyphenols, zinc may also influence matrix organisation, film swelling, and the accessibility of extract-derived antioxidant compounds during the assay. Accordingly, the TEAC values likely reflect not only the total phenolic content incorporated into the films, but also the extent to which these antioxidant species remained available or extractable under the test conditions. The higher TEAC values observed in some zinc-containing formulations may therefore be related to changes in film structure that facilitated the release or accessibility of antioxidant constituents during analysis.

It should also be noted that both DPPH and ABTS assays evaluate radical scavenging activity in simplified chemical systems and do not necessarily represent antioxidant performance in real food matrices. In particular, these assays are more relevant to hydrophilic radical-scavenging behaviour, whereas oxidation in foods often involves more complex pathways, including lipid oxidation [80]. Therefore, although the present results confirm that TE-containing films possess measurable antioxidant activity, their practical effectiveness in specific food systems should be further evaluated using appropriate food-based oxidation models.

#### 3.3.7. Antimicrobial Activity of Composite Films

The antimicrobial activity of the composite films against *Escherichia coli* (Gram-negative) and *Staphylococcus aureus* (Gram-positive) was evaluated using the agar disc diffusion method, and the results are presented in Table 5 and Figure S20.

The control film (T0Z0) and the TE-only formulations (T1Z0–T3Z0) did not produce measurable inhibition zones against either test organism. Although chitosan and plant-derived phenolics are both reported to possess antimicrobial activity, their effectiveness in a disc diffusion assay depends not only on intrinsic activity but also on their ability to diffuse from the film matrix into the surrounding agar medium. Under the present test conditions, the immobilised chitosan matrix and extract-derived constituents may have shown limited diffusivity, which could explain the absence of visible inhibition zones for these formulations [81].

In contrast, zinc-containing films exhibited clear antimicrobial activity, indicating that zinc played the dominant role in the disc-diffusion response. In light of the structural results discussed in Section 3.3.1, this activity is more plausibly attributed to diffusible zinc-containing species released from the film matrix than to direct contact with retained crystalline ZnO particles. The inhibition zones generally increased with zinc loading, which is consistent with a diffusion-mediated antimicrobial effect. Zinc species have been reported to interfere with microbial metabolism, enzyme activity, and membrane-associated functions, and may also contribute indirectly to oxidative stress in bacterial cells [80,82].

At the lowest zinc level (0.1% initial ZnO loading), formulations T0Z1, T1Z1, and T2Z1 produced measurable inhibition zones against *E. coli* (11.3–14.6 mm), whereas no clear inhibition was observed against *S. aureus* under the same conditions. Interestingly, T3Z1 (0.3% TE, 0.1% ZnO) did not produce measurable inhibition zones against either organism. This result suggests that, at high TE content and low zinc loading, interactions between TE-derived polyphenols and zinc-containing species may have reduced the fraction of diffusible antimicrobial species available to migrate into the agar medium. This interpretation is consistent with the structural evidence indicating stronger interactions and greater heterogeneity in films containing both high TE and zinc contents [83].

When the zinc loading was increased to 0.2% and 0.3%, antimicrobial activity was observed against both microorganisms, including in the TE-rich formulations T3Z2 and T3Z3. Under these conditions, the total amount of zinc available may have been sufficient to maintain an effective diffusible fraction despite concurrent interactions with extract-derived constituents. Overall, films containing ≥0.2% initial ZnO inhibited both test organisms, with inhibition zones ranging from 17.1 to 22.7 mm for *E. coli* and from 14.4 to 20.7 mm for *S. aureus*. The generally larger inhibition zones observed for *E. coli* suggest that the two organisms differed in their susceptibility under the present conditions, possibly because of differences in cell envelope structure and permeability [84].

From a formulation perspective, films containing low-to-moderate TE together with zinc, such as T1Z2 and T1Z3, retained antimicrobial performance comparable to that of the zinc-only films while also offering antioxidant functionality. This indicates that both activities may be combined within the same chitosan-based film when the relative TE and zinc contents are appropriately balanced. It should be noted, however, that the disc diffusion assay provides a diffusion-based and semi-quantitative assessment of antimicrobial performance. More rigorous evaluation, including zinc-release measurements and broth-based MIC or MBC assays, would be valuable in future work to clarify the dose–response relationship and the antimicrobial mechanism of these film systems.

#### 3.3.8. Preliminary Application: Visual Assessment of Packaged Bananas

**Figure 8:**
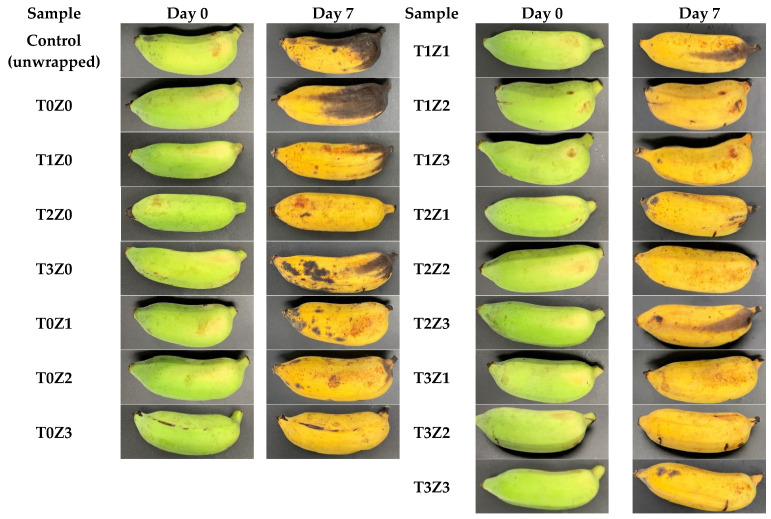
Visual appearance of Namwa bananas (*Musa* ABB group) wrapped with chitosan-based films containing varying TE and ZnO concentrations after storage at ambient temperature (25 ± 2 °C) for 0 and 7 days. Control: unwrapped banana.

To preliminarily evaluate the practical potential of the active films, a visual assessment was conducted using bananas as a perishable model system during 7 days of storage at ambient temperature (Figure 8). This experiment was intended only as an initial qualitative demonstration of film performance and did not include quantitative postharvest measurements.

As expected, the unpackaged control bananas showed substantial visual deterioration by day 7, including pronounced peel browning, senescence spotting, and visible shrivelling. Bananas wrapped with the control chitosan film (T0Z0) appeared to show a slight reduction in browning relative to the unpackaged control. However, noticeable peel dehydration and softening were still observed, which is consistent with the comparatively limited barrier performance of the unmodified oligomer-grade chitosan film.

Bananas wrapped with the zinc-containing formulation T0Z3 showed better visual preservation than those wrapped with T0Z0. After 7 days, these samples retained a greener peel appearance and showed fewer senescence spots. This behaviour is consistent with the lower moisture permeability and more structured matrix observed for the zinc-containing films in the physicochemical analyses. Likewise, bananas wrapped with the TE-only film (T3Z0) appeared to show some reduction in visible browning compared with the control, although the overall visual protection remained limited.

Among the tested formulations, T3Z3 showed the most favourable visual appearance at the end of storage, with less extensive browning and better overall surface integrity than the other groups. This behaviour may reflect the combined contribution of the lower water vapour transmission, darker light-shielding film appearance, and the presence of antioxidant and antimicrobial constituents in the film system. However, because the present assessment was based solely on visual observation, the relative contribution of each factor cannot be determined from this experiment alone.

From a practical standpoint, the results also highlight a trade-off between protective function and product visibility. Although darker and more opaque formulations such as T3Z3 may provide better shielding from light exposure, they also reduce direct visual inspection of the packaged product. Accordingly, such films may be more suitable for applications in which light protection is desirable, whereas intermediate formulations with lower opacity may be preferable when product visibility is important.

It should be emphasised that the present banana study was strictly qualitative and preliminary in nature. No quantitative measurements of weight loss, firmness, peel colour coordinates, microbial load, titratable acidity, total soluble solids, respiration rate, or package headspace composition were performed. Therefore, the present results should be interpreted as an initial visual indication of potential packaging performance rather than definitive evidence of shelf-life extension. More rigorous postharvest studies using quantitative quality parameters will be required to validate the practical efficacy of these films.

## 4. Conclusions

This study investigated the structural and functional behaviour of chitosan-based active packaging films containing *Terminalia catappa* extract (TE) and zinc oxide under acidic casting conditions. The combined XRD, FTIR, SEM, and EDX results indicated that the original crystalline ZnO phase was no longer detectable in the dried films, whereas zinc remained present within the film matrix. These findings are consistent with acid-mediated dissolution and/or structural transformation of ZnO during film preparation, leading to zinc-containing species that interacted with the chitosan-based network.

In the zinc-containing films, these interactions were associated with substantial changes in film structure and performance, including increased tensile strength, reduced moisture content, improved water vapour barrier properties, and higher glass transition temperature. TE contributed antioxidant activity, darker film colour, and reduced light transmittance, whereas films containing both TE and zinc showed more complex behaviour that depended on formulation. In particular, formulations containing both components combined antioxidant and antimicrobial activity with improved mechanical and barrier properties, although high additive loadings also increased opacity and reduced film flexibility.

The antimicrobial results indicated that zinc-containing films were active against *E. coli* and *S. aureus* in the disc diffusion assay, whereas the antioxidant results confirmed that TE was the principal source of radical scavenging activity. The preliminary banana-wrapping study further suggested that selected formulations may provide useful protective effects in practical packaging applications. However, this application test was qualitative in nature and should be interpreted only as an initial visual demonstration rather than definitive evidence of shelf-life extension.

Overall, the results show that the functional performance of these films was governed not only by the presence of TE and zinc, but also by their interactions within the chitosan matrix. This study therefore provides useful insight into the design of chitosan-based active films containing plant-derived antioxidants and zinc-based functional components. Future work should focus on zinc and phenolic migration, quantitative postharvest evaluation, food-specific oxidation studies, oxygen permeability, and long-term film stability in order to further assess the applicability of these materials in food packaging systems [85,86].

## Figures and Tables

**Table 1:** Zinc content (wt% and at%) of chitosan-based films determined by energy-dispersive X-ray spectroscopy (EDX). Values represent map sum spectrum analysis. n.d. = not detected (<0.05 wt%).

Sample ID	Zn	Zn	Sample ID	Zn	Zn	Sample ID	Zn	Zn	Sample ID	Zn	Zn
	(wt%)	(at%)		(wt%)	(at%)		(wt%)	(at%)		(wt%)	(at%)
T0Z0	n.d.	n.d.	T1Z0	n.d.	n.d.	T2Z0	n.d.	n.d.	T3Z0	n.d.	n.d.
T0Z1	6.37	1.42	T1Z1	5.32	1.17	T2Z1	4.28	0.94	T3Z1	5.16	1.15
T0Z2	11.13	2.58	T1Z2	9.91	2.28	T2Z2	11.44	2.67	T3Z2	9.52	2.20
T0Z3	15.08	3.62	T1Z3	16.26	3.95	T2Z3	14.74	3.56	T3Z3	14.79	3.57

**Table 2:** Physical, mechanical, and optical properties of chitosan-based active packaging films.

Sample ID	L*	a*	b*	ΔE*	Haze (%)	Transmittance (%)	Thickness (mm)	Tensile Strength (MPa)	Elongation at Break (%)
T0Z0	83.3 ± 0.6 ^b^	−0.30 ± 0.01 ^e^	26 ± 2 ^c^	Standard	54.7 ± 1.8 ^f^	86.9 ± 0.8 ^a^	0.072 ± 0.013 ^i^	6.0 ± 0.5 ^f^	21.4 ± 0.9 ^cdef^
T1Z0	58 ± 2 ^c^	16 ± 2 ^c^	46 ± 2 ^a^	35.7 ± 1.7 ^e^	62.2 ± 1.2 ^d^	58 ± 4 ^b^	0.080 ± 0.012 ^hi^	5.4 ± 1.6 ^f^	29.1 ± 0.8 ^bc^
T2Z0	48 ± 2 ^d^	19.9 ± 0.6 ^a^	35 ± 2 ^b^	41.1 ± 1.5 ^d^	71.1 ± 1.6 ^c^	43 ± 4 ^de^	0.088 ± 0.010 ^fgh^	8.7 ± 1.4 ^f^	29 ± 3 ^bc^
T3Z0	43 ± 2 ^fg^	19.4 ± 0.3 ^a^	26 ± 4 ^c^	45 ± 2 ^bc^	78.6 ± 0.3 ^b^	34 ± 2 ^g^	0.100 ± 0.014 ^def^	5.8 ± 0.8 ^f^	22 ± 4 ^cde^
T0Z1	86.8 ± 0.1 ^a^	−1.0 ± 0.1 ^e^	12.0 ± 0.4 ^d^	16.2 ± 1.8 ^g^	42.0 ± 0.6 ^g^	88.9 ± 0.4 ^a^	0.082 ± 0.010 ^hi^	15.7 ± 1.9 ^e^	28.5 ± 1.8 ^bc^
T0Z2	87.5 ± 0.2 ^a^	−0.8 ± 0.1 ^e^	7.9 ± 0.8 ^e^	19.3 ± 0.8 ^f^	41.5 ± 1.8 ^g^	89.7 ± 0.1 ^a^	0.087 ± 0.015 ^gh^	27 ± 3 ^cd^	33 ± 5 ^bcd^
T0Z3	85.3 ± 0.2 ^ab^	0.3 ± 0.1 ^e^	14.3 ± 0.3 ^d^	12.7 ± 0.3 ^h^	29 ± 2 ^h^	88.9 ± 0.4 ^a^	0.10 ± 0.02 ^efg^	36 ± 4 ^a^	24 ± 4 ^cde^
T1Z1	58.2 ± 1.1 ^c^	15.5 ± 0.7 ^cd^	47.8 ± 0.8 ^a^	36.3 ± 0.5 ^e^	58.1 ± 0.6 ^e^	57.4 ± 1.4 ^b^	0.086 ± 0.010 ^gh^	18 ± 3 ^e^	37 ± 4 ^a^
T1Z2	58.3 ± 1.0 ^c^	14.3 ± 0.6 ^d^	45.7 ± 0.3 ^a^	34.5 ± 1.0 ^e^	58.8 ± 0.9 ^e^	53 ± 2 ^c^	0.090 ± 0.011 ^fgh^	29.4 ± 1.2 ^bc^	32 ± 3 ^ab^
T1Z3	56.8 ± 0.9 ^c^	14.3 ± 0.6 ^d^	44.3 ± 0.1 ^a^	34.9 ± 0.9 ^e^	59.5 ± 1.1 ^e^	52.9 ± 0.7 ^c^	0.092 ± 0.016 ^fgh^	33 ± 4 ^ab^	16 ± 2 ^efg^
T2Z1	46.1 ± 1.4 ^de^	19.5 ± 0.8 ^a^	32 ± 4 ^b^	42 ± 2 ^cd^	72.4 ± 0.9 ^c^	44.3 ± 1.2 ^d^	0.106 ± 0.015 ^cde^	14.8 ± 1.7 ^e^	28 ± 4 ^bcd^
T2Z2	44 ± 3 ^ef^	19.4 ± 0.1 ^a^	31.2 ± 1.5 ^b^	42.5 ± 0.8 ^cd^	71.9 ± 0.7 ^c^	40 ± 2 ^ef^	0.107 ± 0.012 ^cde^	23.9 ± 1.1 ^d^	20 ± 3 ^def^
T2Z3	42.8 ± 1.4 ^efg^	17.6 ± 0.8 ^b^	26 ± 2 ^c^	44.2 ± 1.0 ^bc^	71.5 ± 0.4 ^c^	38.4 ± 1.4 ^f^	0.114 ± 0.010 ^bc^	30 ± 3 ^bc^	14 ± 4 ^fgh^
T3Z1	40.2 ± 1.4 ^g^	17.8 ± 0.1 ^b^	23 ± 2 ^c^	46.8 ± 1.5 ^b^	78.3 ± 0.3 ^b^	32.9 ± 1.5 ^g^	0.136 ± 0.011 ^a^	14 ± 2 ^e^	23 ± 4 ^cde^
T3Z2	34.4 ± 0.3 ^h^	16.8 ± 0.2 ^bc^	13.5 ± 0.5 ^d^	53.3 ± 0.3 ^a^	79.6 ± 0.8 ^ab^	31 ± 3 ^g^	0.111 ± 0.015 ^cd^	27 ± 2 ^cd^	10 ± 5 ^gh^
T3Z3	36.2 ± 1.1 ^h^	16.0 ± 0.1 ^c^	16.2 ± 1.4 ^d^	50.8 ± 1.3 ^a^	81.5 ± 0.7 ^a^	21.5 ± 1.5 ^h^	0.123 ± 0.015 ^b^	27 ± 4 ^cd^	7 ± 2 ^h^

All formulations contained 1.5% (*w*/*v*) chitosan, 0.5% (*v*/*v*) glycerol, and 1.0% (*v*/*v*) acetic acid. Values are mean ± SD (*n* = 3). Different superscript letters within the same column indicate significant differences (*p* < 0.05). ΔE* was calculated using T0Z0 as the reference standard.

**Table 3:** Thermal properties of chitosan-based films: glass transition temperature (T_g_), melting temperature (T_m_), total weight loss (%), and residual weight at 800 °C (%) determined by DSC and TGA.

Sample ID	DSC		TGA	
	T_g_ (°C)	T_m_ (°C)	Weight Loss (%)	Residue (%)
T0Z0	74.5	146.6	82.3	17.7
T1Z0	75.5	166.2	84.3	15.7
T2Z0	78.0	163.3	85.0	15.0
T3Z0	78.2	164.6	78.5	21.5
T0Z1	72.4	155.7	76.9	23.1
T0Z2	83.0	153.1	73.6	26.4
T0Z3	103.3	156.9	73.0	27.0
T1Z1	75.9	153.1	77.8	22.2
T1Z2	100.0	159.1	72.6	27.4
T1Z3	110.7	153.8	71.0	29.0
T2Z1	105.6	156.4	77.3	22.7
T2Z2	111.1	159.3	70.5	29.5
T2Z3	117.9	151.3	68.3	31.8
T3Z1	91.0	159.9	73.0	27.0
T3Z2	111.7	165.8	70.4	29.6
T3Z3	122.9	166.4	68.9	31.2

All formulations contained 1.5% (*w*/*v*) chitosan, 0.5% (*v*/*v*) glycerol, and 1.0% (*v*/*v*) acetic acid. T_g_ = glass transition temperature; T_m_ = melting temperature. Weight loss and residue were determined at 800 °C.

**Table 4:** Moisture content (MC), water vapour transmission rate (WVTR), water vapour permeability (WVP), and water contact angle (CA) of chitosan-based films incorporated with TE and ZnO.

Sample ID	Moisture Content (%)	WVTR (g/(m^2^·h))	WVP (×10^−8^ g·cm/(cm^2^·day·Pa))	Contact Angle (°)
T0Z0	19.8 ± 0.6 ^e^	2.16 ± 0.07 ^a^	1.41 ± 0.03 ^ab^	92 ± 9 ^a^
T1Z0	18.6 ± 0.5 ^de^	2.08 ± 0.09 ^ab^	1.48 ± 0.07 ^ab^	88 ± 5 ^a^
T2Z0	18.6 ± 1.2 ^de^	2.09 ± 0.08 ^ab^	1.45 ± 0.09 ^ab^	80 ± 4 ^a^
T3Z0	17.7 ± 0.7 ^d^	2.08 ± 0.09 ^ab^	1.70 ± 0.08 ^a^	71 ± 8 ^a^
T0Z1	15.54 ± 0.14 ^c^	2.05 ± 0.13 ^ab^	1.12 ± 0.07 ^cde^	79 ± 9 ^a^
T0Z2	13.3 ± 0.3 ^ab^	2.04 ± 0.03 ^ab^	1.218 ± 0.004 ^bcde^	89 ± 9 ^a^
T0Z3	12.7 ± 0.3 ^ab^	1.81 ± 0.18 ^ab^	1.13 ± 0.12 ^cde^	85 ± 3 ^a^
T1Z1	14.6 ± 0.9 ^bc^	2.04 ± 0.14 ^ab^	1.20 ± 0.15 ^bcde^	87 ± 3 ^a^
T1Z2	13.4 ± 0.4 ^ab^	2.0 ± 0.2 ^ab^	1.01 ± 0.12 ^e^	86.0 ± 1.7 ^a^
T1Z3	13.1 ± 0.2 ^ab^	2.02 ± 0.17 ^ab^	1.32 ± 0.06 ^bcd^	94 ± 3 ^a^
T2Z1	14.2 ± 1.2 ^bc^	2.00 ± 0.17 ^ab^	1.11 ± 0.15 ^de^	70 ± 9 ^a^
T2Z2	13.4 ± 0.5 ^ab^	1.98 ± 0.18 ^ab^	1.4 ± 0.2 ^ab^	89 ± 4 ^a^
T2Z3	12.2 ± 0.3 ^a^	1.8 ± 0.3 ^ab^	1.4 ± 0.2 ^bc^	79 ± 2 ^a^
T3Z1	15.33 ± 0.17 ^c^	2.0 ± 0.2 ^ab^	1.44 ± 0.11 ^ab^	70 ± 9 ^a^
T3Z2	13.3 ± 0.2 ^ab^	1.97 ± 0.14 ^ab^	1.5 ± 0.1 ^ab^	78 ± 10 ^a^
T3Z3	12.0 ± 0.7 ^a^	1.7 ± 0.3 ^b^	1.48 ± 0.09 ^ab^	88 ± 7 ^a^

All formulations contained 1.5% (*w*/*v*) chitosan, 0.5% (*v*/*v*) glycerol, and 1.0% (*v*/*v*) acetic acid. Values are mean ± SD (*n* = 3). Different superscript letters within the same column indicate significant differences (*p* < 0.05) by Tukey’s honestly significant difference (HSD) test. Contact angle was measured at t = 0 s using the sessile drop method.

**Table 5:** Antimicrobial activity (inhibition zone diameter, mm) of chitosan-based films against *Escherichia coli* and *Staphylococcus aureus* determined by disc diffusion method. Film disc diameter = 10 mm. Values are expressed as mean ± SD (*n* = 3). “0 ^e^” indicates no detectable inhibition zone. Different superscript letters within each column indicate significant differences (*p* < 0.05).

Sample ID	Inhibition Zone (mm)	
	*E. coli* (Gram-Negative)	*S. aureus* (Gram-Positive)
T0Z0	0 ^e^	0 ^d^
T1Z0	0 ^e^	0 ^d^
T2Z0	0 ^e^	0 ^d^
T3Z0	0 ^e^	0 ^d^
T0Z1	11.3 ± 1.6 ^d^	0 ^d^
T0Z2	19.3 ± 1.8 ^ab^	16.8 ± 1.0 ^b^
T0Z3	22 ± 3 ^a^	21 ± 2 ^a^
T1Z1	14.6 ± 1.2 ^cd^	0 ^d^
T1Z2	20 ± 2 ^ab^	17.3 ± 0.9 ^b^
T1Z3	23 ± 2 ^a^	20.6 ± 0.1 ^a^
T2Z1	14.2 ± 0.8 ^cd^	0 ^d^
T2Z2	18 ± 3 ^bc^	15.5 ± 1.0 ^bc^
T2Z3	20 ± 2 ^ab^	19.9 ± 1.4 ^a^
T3Z1	0 ^e^	0 ^d^
T3Z2	17 ± 3 ^bc^	14 ± 2 ^c^
T3Z3	20.8 ± 1.7 ^ab^	19.5 ± 0.4 ^a^

All formulations contained 1.5% (*w*/*v*) chitosan, 0.5% (*v*/*v*) glycerol, and 1.0% (*v*/*v*) acetic acid. Different superscript letters within the same column indicate significant differences (*p* < 0.05) by Tukey’s honestly significant difference (HSD) test.

## Data Availability

The original contributions presented in this study are included in the article/Supplementary Materials. Further inquiries can be directed to the corresponding author.

## Supplementary Materials

The following supporting information can be downloaded at: https://www.mdpi.com/article/10.3390/polym18080928/s1, Table S1: Composition of film-forming solutions; Table S2: Extraction yields of *Terminalia catappa* leaf extract (TE) under various microwave-assisted extraction conditions (power and time) and drying techniques, expressed on a dry-weight basis; Table S3: Total phenolic content (TPC) and antioxidant activity (DPPH and ABTS) of *Terminalia catappa* leaf extracts prepared under various microwave-assisted extraction conditions and drying techniques; Table S4: Full elemental composition (C, O, Na, Ca, Zn; wt% and at%) of all 16 chitosan-based film formulations determined by EDX map sum spectrum analysis; Table S5: Trolox equivalent antioxidant capacity (TEAC) of chitosan-based films incorporated with *Terminalia catappa* leaf extract (TE) and ZnO; Table S6: Summary of linear fitting of Debye plots and calculated weight-average molecular weight (Mw) of chitosan samples in 0.1 M acetic acid/0.2 M NaCl at 25 °C; Figure S1: UV–Vis absorption spectra (250–600 nm) of *T. catappa* leaf extracts prepared at (a) 130 W with freeze drying, (b) 440 W with freeze drying, (c) 130 W with vacuum drying, and (d) 440 W with vacuum drying, at extraction times of 5, 10, 15, and 20 min; Figure S2: FTIR spectra (4000–400 cm^−1^) of *T. catappa* leaf extracts prepared at (a) 130 W with freeze drying, (b) 440 W with freeze drying, (c) 130 W with vacuum drying, and (d) 440 W with vacuum drying, at extraction times of 5, 10, 15, and 20 min; Figure S3: SEM micrographs and EDX elemental maps of T0Z0 film formulations; Figure S4: SEM micrographs and EDX elemental maps of T1Z0 film formulations; Figure S5: SEM micrographs and EDX elemental maps of T2Z0 film formulations; Figure S6: SEM micrographs and EDX elemental maps of T3Z0 film formulations; Figure S7: SEM micrographs and EDX elemental maps of T0Z1 film formulations; Figure S8: SEM micrographs and EDX elemental maps of T0Z2 film formulations; Figure S9: SEM micrographs and EDX elemental maps of T0Z3 film formulations; Figure S10: SEM micrographs and EDX elemental maps of T1Z1 film formulations; Figure S11: SEM micrographs and EDX elemental maps of T1Z2 film formulations; Figure S12: SEM micrographs and EDX elemental maps of T1Z3 film formulations; Figure S13: SEM micrographs and EDX elemental maps of T2Z1 film formulations; Figure S14: SEM micrographs and EDX elemental maps of T2Z2 film formulations; Figure S15: SEM micrographs and EDX elemental maps of T2Z3 film formulations; Figure S16: SEM micrographs and EDX elemental maps of T3Z1 film formulations; Figure S17: SEM micrographs and EDX elemental maps of T3Z2 film formulations; Figure S18: SEM micrographs and EDX elemental maps of T3Z3 film formulations; Figure S19: TGA thermograms of chitosan-based active packaging films grouped by TE concentration: (a) 0%, (b) 0.1%, (c) 0.2%, and (d) 0.3% *w*/*v*, with varying ZnO levels (0–0.3% *w*/*v*); Figure S20: Photographs of disc diffusion assay plates showing inhibition zones of chitosan-based films against *E. coli* (a–d) and *S. aureus* (e–h). Film discs were incubated at 37 °C for 24 h; Figure S21: Official Certificate of Analysis (COA) of the commercial food-grade chitosan oligomer (derived from king crab shell, *Paralithodes camtschaticus*) utilized in this study. The document, provided by the supplier (Union Science Co., Ltd., Chiang Mai, Thailand; Lot No. 20211017), corroborates the key physicochemical specifications, specifically indicating a low viscosity (<5 cps) and a high degree of deacetylation (92.00%); Figure S22: Debye plots of chitosan samples obtained from two independent concentration series in 0.1 M acetic acid/0.2 M NaCl at 25 °C. (A) Series A and (B) Series B.

## Abbreviations

The following abbreviations are used in this manuscript:

ABTS	2,2′-Azino-bis(3-ethylbenzothiazoline-6-sulfonic acid)
ANOVA	Analysis of variance
ATR	Attenuated total reflectance
CA	Contact angle
CFU	Colony-forming units
DLS	Dynamic light scattering
DPPH	2,2-Diphenyl-1-picrylhydrazyl
DSC	Differential scanning calorimetry
EAB	Elongation at break
EDX	Energy-dispersive X-ray spectroscopy
Eg	Optical band gap
FC	Folin–Ciocalteu
FD	Freeze drying
FTIR	Fourier-transform infrared spectroscopy
FWHM	Full width at half maximum
GAE	Gallic acid equivalents
HAT	Hydrogen atom transfer
HSD	Honestly significant difference
JCPDS	Joint Committee on Powder Diffraction Standards
KBr	Potassium bromide
KOH	Potassium hydroxide
MAE	Microwave-assisted extraction
MC	Moisture content
MIC	Minimum inhibitory concentration
MW	Molecular weight
NA	Nutrient agar
NPs	Nanoparticles
PTFE	Polytetrafluoroethylene
ROS	Reactive oxygen species
SD	Standard deviation
SEM	Scanning electron microscopy
SET	Single electron transfer
TE	*Terminalia catappa* leaf extract
TEAC	Trolox equivalent antioxidant capacity
Tg	Glass transition temperature
TGA	Thermogravimetric analysis
Tm	Melting temperature
TPC	Total phenolic content
TS	Tensile strength
UV–Vis	Ultraviolet–visible spectroscopy
VD	Vacuum drying
WVP	Water vapour permeability
WVTR	Water vapour transmission rate
XRD	X-ray diffraction
ZnO	Zinc oxide
