# Supplementary material for: Chitosan-Based Active Packaging Films Incorporating Terminalia catappa Leaf Extract and Zinc Oxide Precursors for Sustainable Food Packaging
Source: Polymers (Basel). 2026 Apr 10;18(8):928. doi: 10.3390/polym18080928 (PMC13119734; doi:10.3390/polym18080928)
Supplement: Supplementary file 1 [file polymers-18-00928-s001.zip › polymers-4237402-supplementary.pdf]

Supplementary Materials
Chitosan-Based Active Packaging Films Incorporating *Terminalia catappa* Leaf Extract and Zinc Oxide for Sustainable Food Packaging

2. Materials and Methods

Table S1. Composition of film-forming solutions

Sample ID	*T. catappa* leaf extract % (w/v)	Zinc Oxide % (w/v)
T0Z0	0.0	0.0
T1Z0	0.1	0.0
T2Z0	0.2	0.0
T3Z0	0.3	0.0
T0Z1	0.0	0.1
T0Z2	0.0	0.2
T0Z3	0.0	0.3
T1Z1	0.1	0.1
T1Z2	0.1	0.2
T1Z3	0.1	0.3
T2Z1	0.2	0.1
T2Z2	0.2	0.2
T2Z3	0.2	0.3
T3Z1	0.3	0.1
T3Z2	0.3	0.2
T3Z3	0.3	0.3

All formulations contained 1.5% (w/v) chitosan, 0.5% (v/v) glycerol, and 1.0% (v/v) acetic acid. Samples were designated as TxZy, where T and Z represent T. catappa leaf extract and ZnO, respectively; x and y denote concentration levels 0–3 corresponding to 0, 0.1, 0.2, and 0.3% (w/v).

3. Results and discussion

3.1.1. Temperature Profile, Extraction Yield, Total Phenolic Content and Antioxidant Activities

Table S2 Extraction yields of *Terminalia catappa* leaf extract (TE) under various microwave-assisted extraction conditions (power and time) and drying techniques, expressed on a dry-weight basis.

Microwave power (Watt)	Time (Min)	Yield (%)	
		Freeze dry technique	Vacuum dry technique
130	5	14.8 ± 1.9f	13.4 ± 0.1^{f}
	10	18.2 ± 1.6^{e}	16.6 ± 0.3^{ef}
	15	19.3 ± 1.0^{de}	17.4 ± 0.4^{e}
	20	19.4 ± 1.4^{de}	18.8 ± 0.3^{e}
440	5	25.6 ± 0.3^{bc}	20.3 ± 1.3^{de}
	10	26.4 ± 0.1^{b}	22.3 ± 0.9^{cd}
	15	28.2 ± 0.3^{ab}	25 ± 2^{bc}
	20	29.5 ± 0.2^{a}	27 ± 2^{b}

Data are expressed as the mean ± standard deviation (n = 3). Different superscript letters within the same column indicate statistically significant differences (p < 0.05) according to Tukey's honestly significant difference (HSD) test.

Table S3 Total phenolic content (TPC) and antioxidant activity (DPPH and ABTS) of Terminalia catappa leaf extracts prepared under various microwave-assisted extraction conditions and drying techniques.

Microwave power (Watt)	Time (min)	TPC (mg GAE/g extract)		DPPH (mg Trolox/g extract)		ABTS (mg Trolox/g extract)	
		Freeze dry	Vacuum dry	Freeze dry	Vacuum dry	Freeze dry	Vacuum dry
130	5	304 ± 16^{g}	134 ± 4^{h}	86 ± 3^{b}	43 ± 4^{d}	130 ± 4^{d}	65 ± 3^{c}
	10	375 ± 11^{f}	329 ± 12^{f}	89.2 ± 1.3^{b}	73 ± 4^{b}	127 ± 9^{d}	95 ± 3^{a}
	15	530 ± 30^{cd}	350 ± 20^{ef}	87 ± 4^{b}	89.0 ± 0.8^{a}	128 ± 9^{d}	86 ± 2^{ab}
	20	530 ± 80^{cd}	132 ± 13^{h}	84 ± 3^{b}	61 ± 4^{c}	152 ± 3^{bc}	57.3 ± 1.3^{cd}
440	5	442 ± 13^{e}	125 ± 10^{h}	93 ± 9^{b}	38.1 ± 1.6^{de}	151 ± 8^{bc}	50 ± 4^{d}
	10	530 ± 20^{cd}	149 ± 14^{h}	96 ± 8^{b}	42 ± 3^{d}	143 ± 11^{cd}	83.3 ± 0.5^{b}
	15	590 ± 20^{bc}	152 ± 8^{gh}	97 ± 7^{b}	42 ± 6^{d}	164.2 ± 0.3^{ab}	86 ± 10^{ab}
	20	640 ± 30^{a}	183 ± 10^{g}	111 ± 3^{a}	35.2 ± 1.1^{e}	175 ± 7^{a}	95 ± 5^{a}

Data are expressed as the mean ± standard deviation (n = 3). Different superscript letters within the same column indicate statistically significant differences ($p < 0.05$) according to Tukey's honestly significant difference (HSD) test.

3.1.2. UV-Vis Spectroscopy

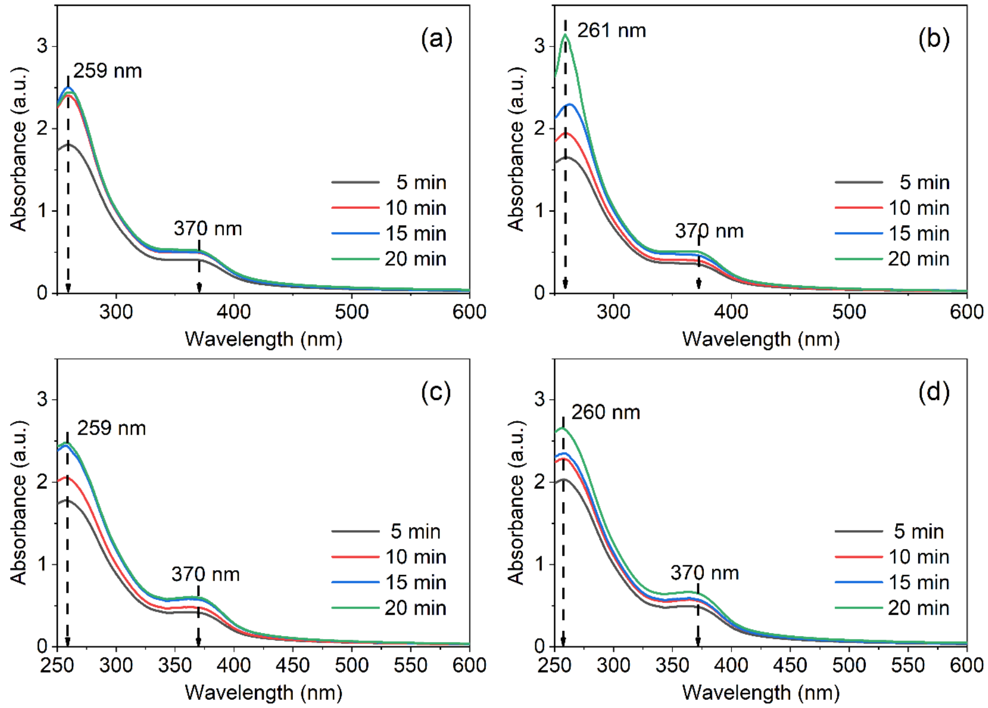

Figure S1. UV-Vis absorption spectra (250–600 nm) of *T. catappa* leaf extracts prepared at (a) 130 W with freeze drying, (b) 440 W with freeze drying, (c) 130 W with vacuum drying, and (d) 440 W with vacuum drying, at extraction times of 5, 10, 15, and 20 min.

3.1.3. FTIR Analysis

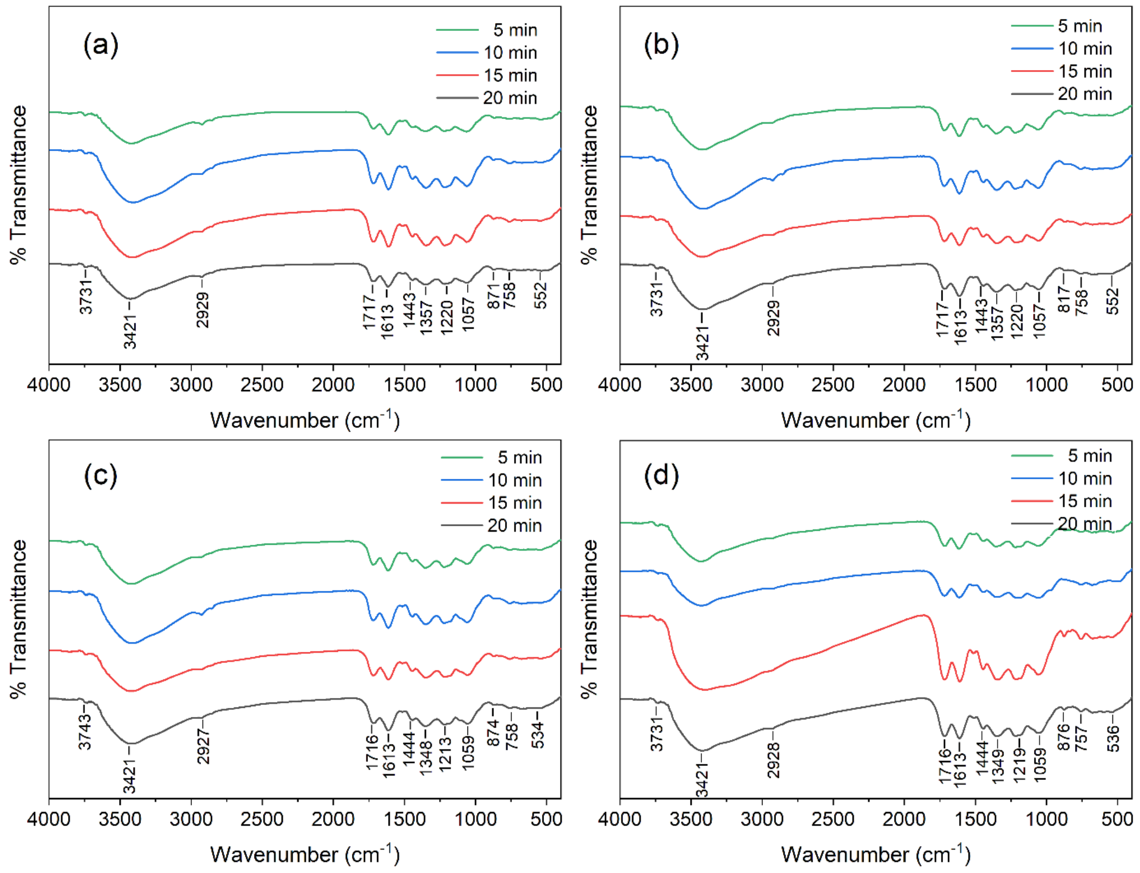

Figure S2. FTIR spectra (4000–400 cm^{-1}) of *T. catappa* leaf extracts prepared at (a) 130 W with freeze drying, (b) 440 W with freeze drying, (c) 130 W with vacuum drying, and (d) 440 W with vacuum drying, at extraction times of 5, 10, 15, and 20 min.

3.3.1. Structural and Morphological Characterization of Films: XRD, FTIR, SEM, and EDX

Table S4. Full elemental composition (C, O, Na, Ca, Zn; wt% and at%) of all 16 chitosan-based film formulations determined by EDX map sum spectrum analysis.

Sample ID	C (wt%)	O (wt%)	Na (wt%)	Ca (wt%)	Zn (wt%)	C (at%)	O (at%)	Na (at%)	Ca (at%)	Zn (at%)
T0Z0	51.29	48.33	0.09	0.24	n.d.	58.48	41.37	0.05	0.08	n.d.
T0Z1	46.35	45.65	1.29	0.33	**6.37**	56.13	41.51	0.82	0.12	**1.42**
T0Z2	44.33	42.31	1.99	0.24	**11.13**	55.93	40.08	1.31	0.09	**2.58**
T0Z3	43.02	38.95	2.63	0.33	**15.08**	56.24	38.22	1.79	0.13	**3.62**
T1Z0	50.49	49.08	0.05	0.38	n.d.	57.72	42.12	0.03	0.13	n.d.
T1Z1	47.05	46.13	1.09	0.40	**5.32**	56.45	41.55	0.68	0.14	**1.17**
T1Z2	44.52	43.08	2.14	0.34	**9.91**	55.72	40.48	1.40	0.13	**2.28**

**Sample ID**	**C (wt%)**	**O (wt%)**	**Na (wt%)**	**Ca (wt%)**	**Zn (wt%)**	**C (at%)**	**O (at%)**	**Na (at%)**	**Ca (at%)**	**Zn (at%)**
T1Z3	43.17	36.51	3.62	0.45	**16.26**	57.11	36.26	2.50	0.18	**3.95**
T2Z0	47.88	50.67	0.31	1.14	n.d.	55.40	44.02	0.18	0.40	n.d.
T2Z1	46.73	46.95	1.21	0.82	**4.28**	55.87	42.14	0.76	0.30	**0.94**
T2Z2	43.65	42.35	1.77	0.79	**11.44**	55.46	40.39	1.17	0.30	**2.67**
T2Z3	41.34	40.70	2.59	0.63	**14.74**	54.29	40.13	1.78	0.25	**3.56**
T3Z0	46.84	51.48	0.36	1.29	n.d.	54.42	44.90	0.22	0.45	n.d.
T3Z1	45.48	47.45	1.00	0.91	**5.16**	54.90	42.99	0.63	0.33	**1.15**
T3Z2	43.07	44.41	2.07	0.93	**9.52**	54.16	41.93	1.36	0.35	**2.20**
T3Z3	41.29	40.21	3.19	0.53	**14.79**	54.32	39.71	2.19	0.21	**3.57**

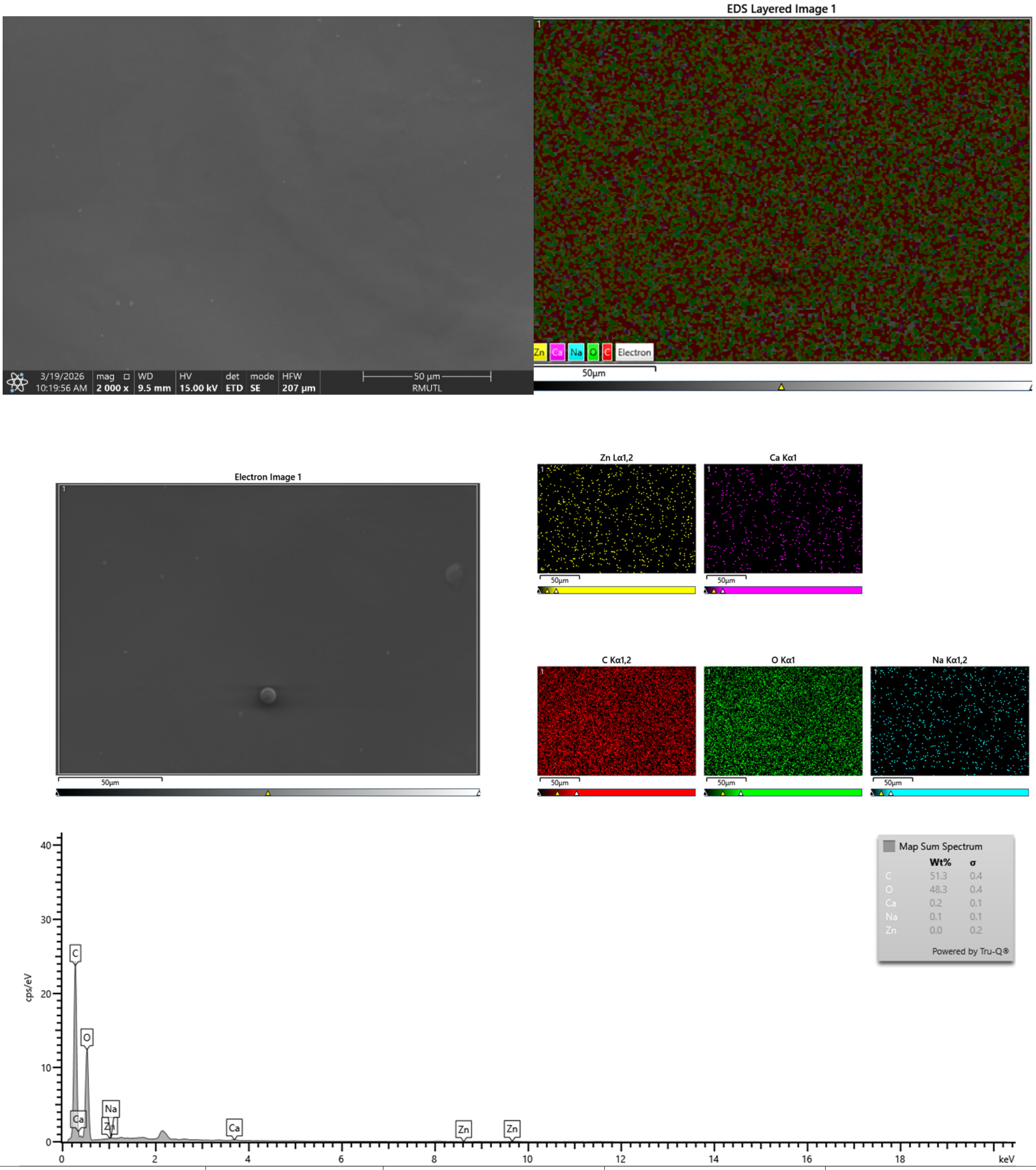

**Map Sum Spectrum**				
**Element**	**Line Type**	**Weight %**	**Weight % Sigma**	**Atomic %**
C	K series	51.29	0.45	58.48
O	K series	48.33	0.45	41.37
Na	K series	0.09	0.08	0.05
Ca	K series	0.24	0.07	0.08
Zn	L series	0.04	0.17	0.01
Total		100.00		100.00

Figure S3. SEM micrographs and EDX elemental maps of T0Z0 film formulations

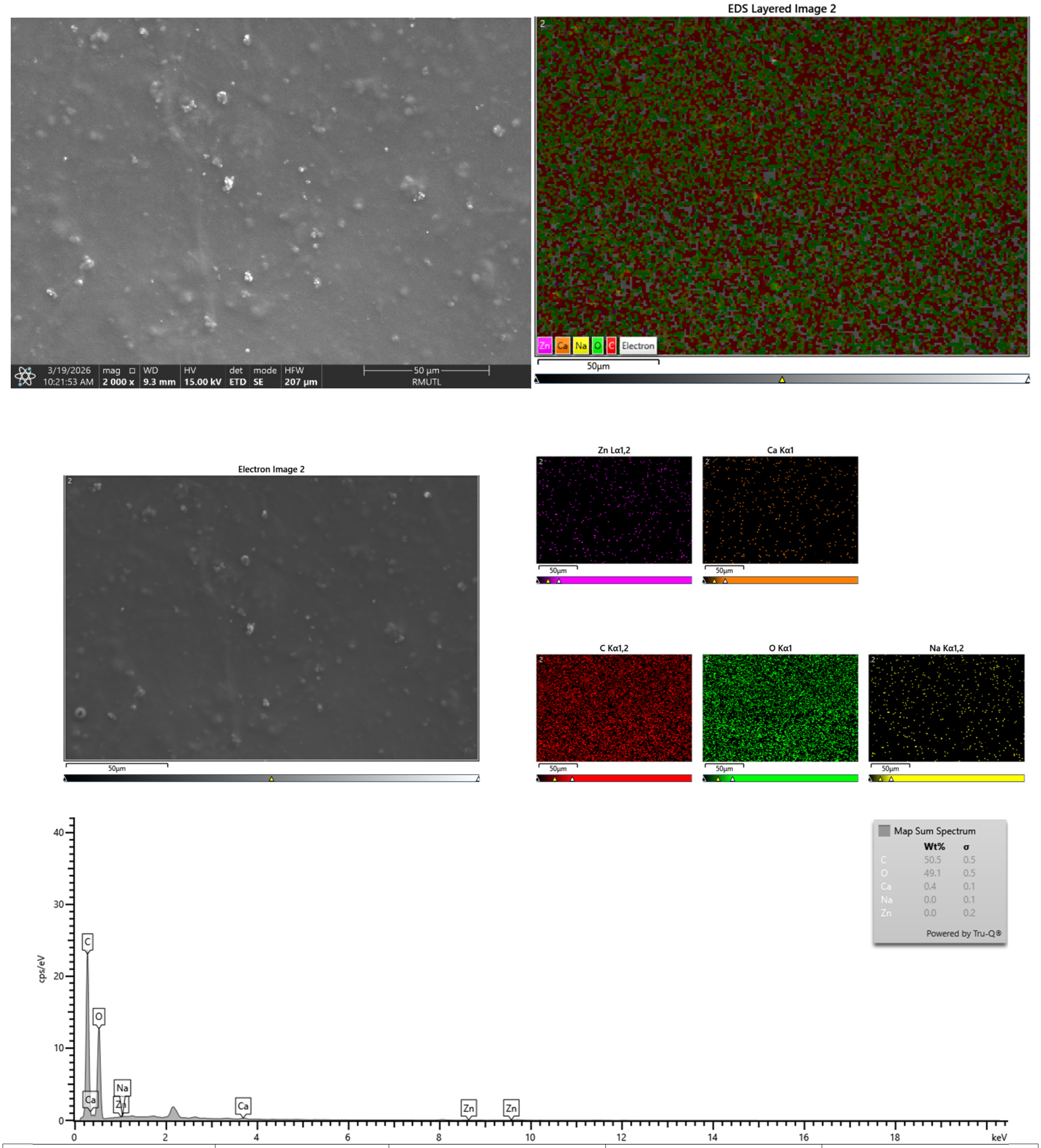

Map Sum Spectrum				
**Element**	**Line Type**	**Weight %**	**Weight % Sigma**	**Atomic %**
C	K series	50.49	0.54	57.72
O	K series	49.08	0.54	42.12
Na	K series	0.05	0.11	0.03
Ca	K series	0.38	0.09	0.13
Zn	L series	0.00	0.22	0.00
Total		100.00		100.00

Figure S4. SEM micrographs and EDX elemental maps of T1Z0 film formulations

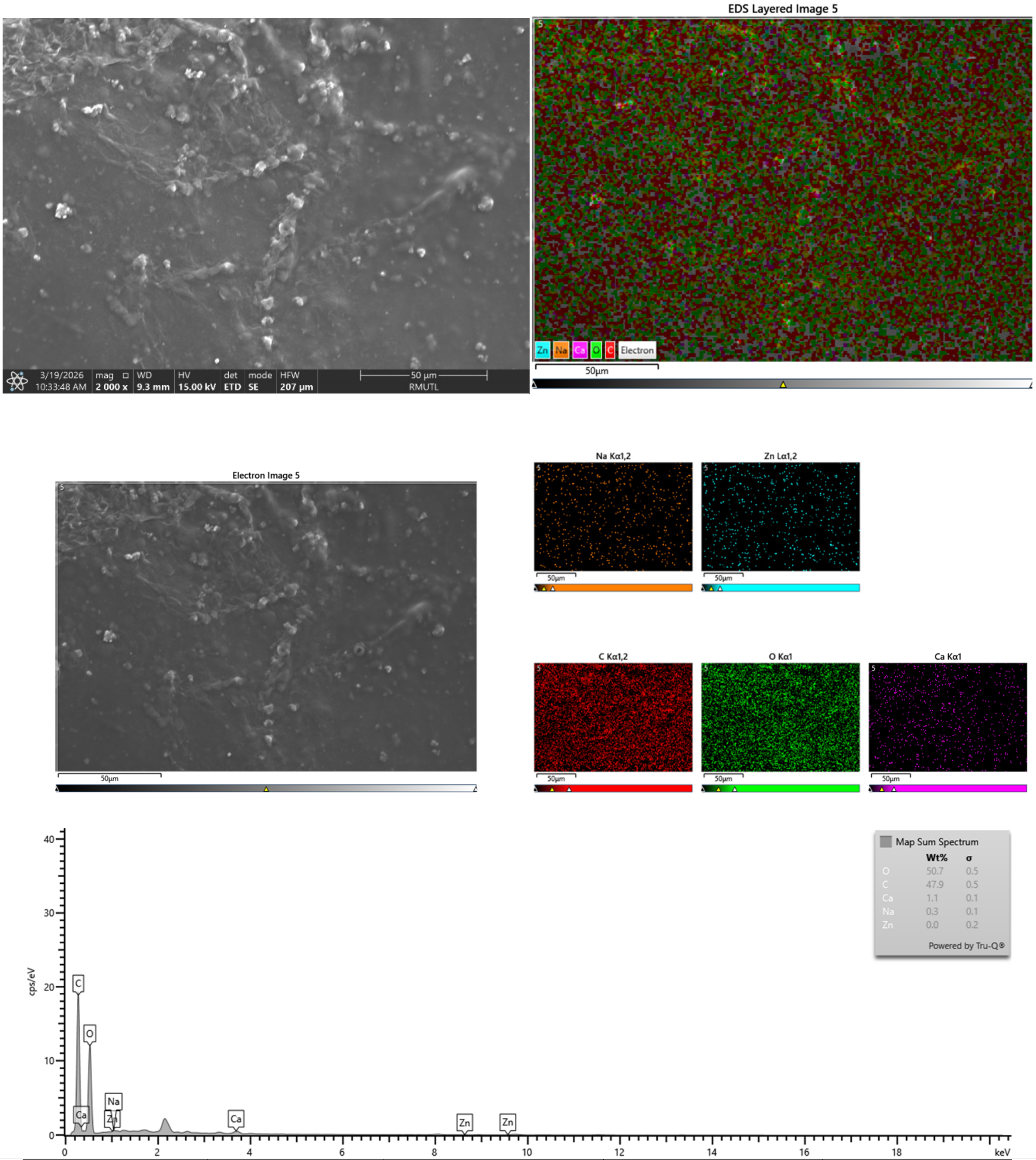

Map Sum Spectrum				
**Element**	**Line Type**	**Weight %**	**Weight % Sigma**	**Atomic %**
C	K series	47.88	0.53	55.40
O	K series	50.67	0.54	44.02
Na	K series	0.31	0.12	0.18
Ca	K series	1.14	0.11	0.40
Zn	L series	0.00	0.24	0.00
Total		100.00		100.00

Figure S5. SEM micrographs and EDX elemental maps of T2Z0 film formulations

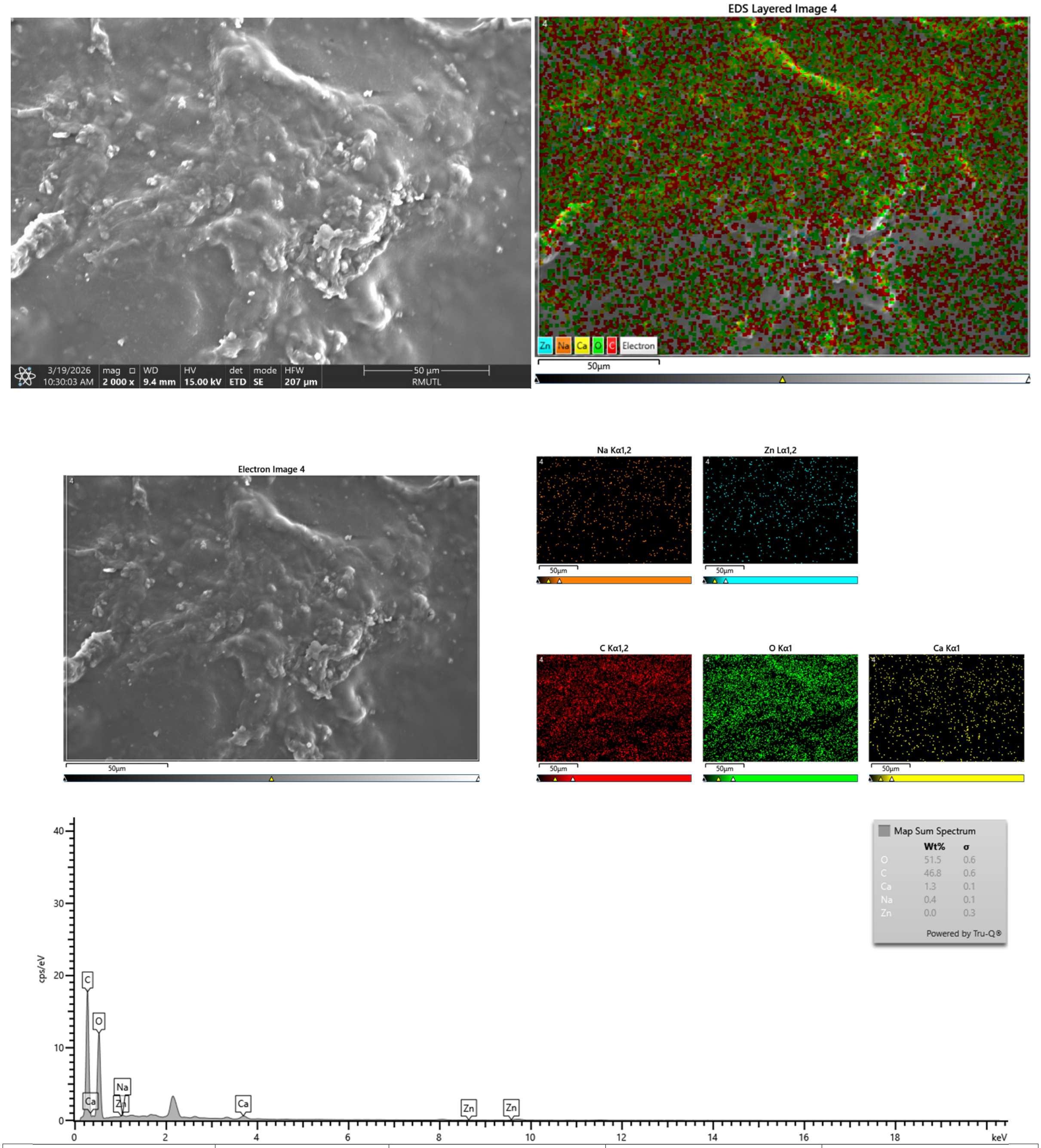

**Map Sum Spectrum**				
**Element**	**Line Type**	**Weight %**	**Weight % Sigma**	**Atomic %**
C	K series	46.84	0.59	54.42
O	K series	51.48	0.60	44.90
Na	K series	0.36	0.14	0.22
Ca	K series	1.29	0.13	0.45
Zn	L series	0.04	0.28	0.01
Total		100.00		100.00

Figure S6. SEM micrographs and EDX elemental maps of T3Z0 film formulations

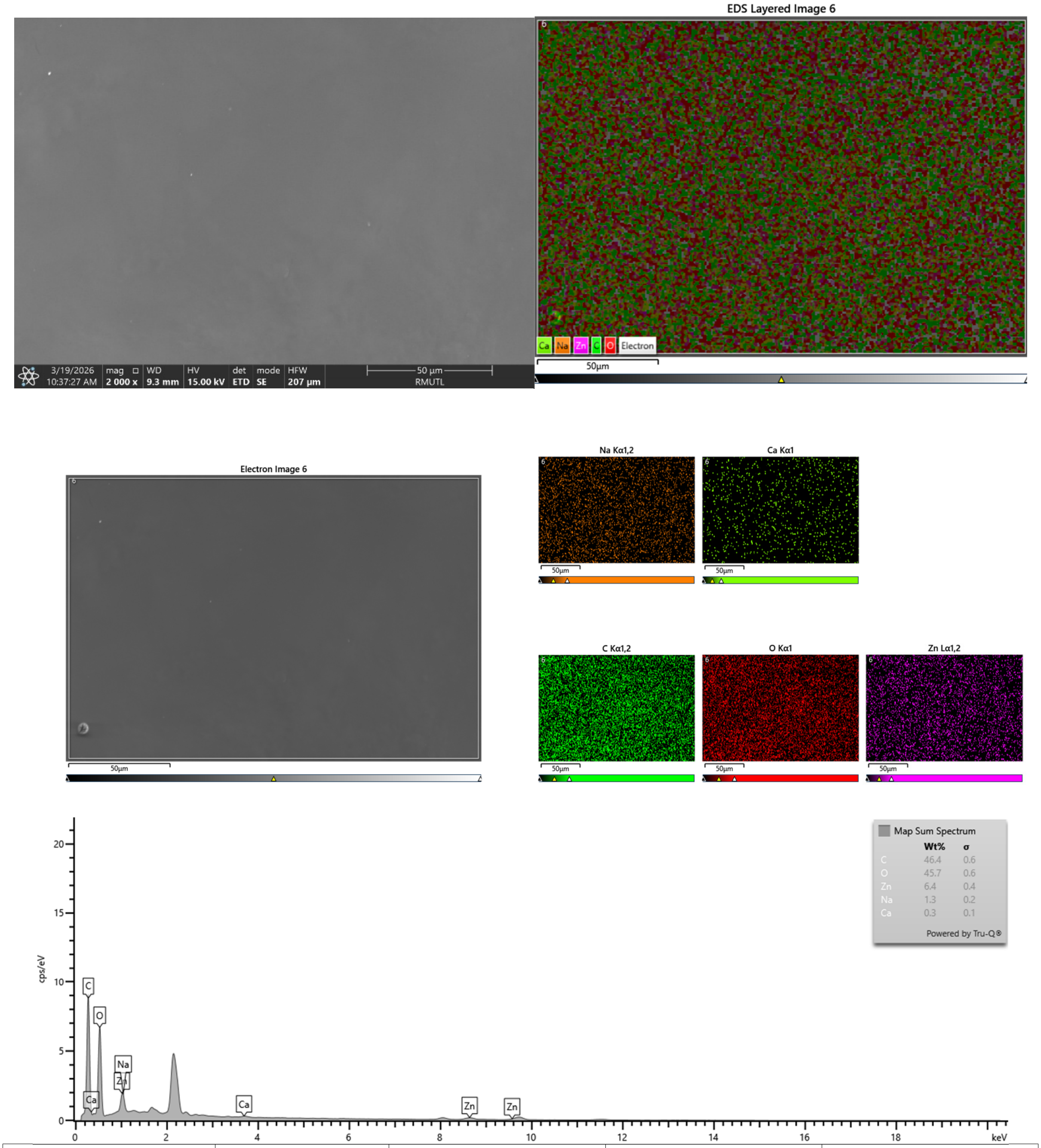

**Map Sum Spectrum**				
**Element**	**Line Type**	**Weight %**	**Weight % Sigma**	**Atomic %**
C	K series	46.35	0.57	56.13
O	K series	45.65	0.57	41.51
Na	K series	1.29	0.23	0.82
Ca	K series	0.33	0.12	0.12
Zn	L series	6.37	0.40	1.42
Total		100.00		100.00

Figure S7. SEM micrographs and EDX elemental maps of T0Z1 film formulations

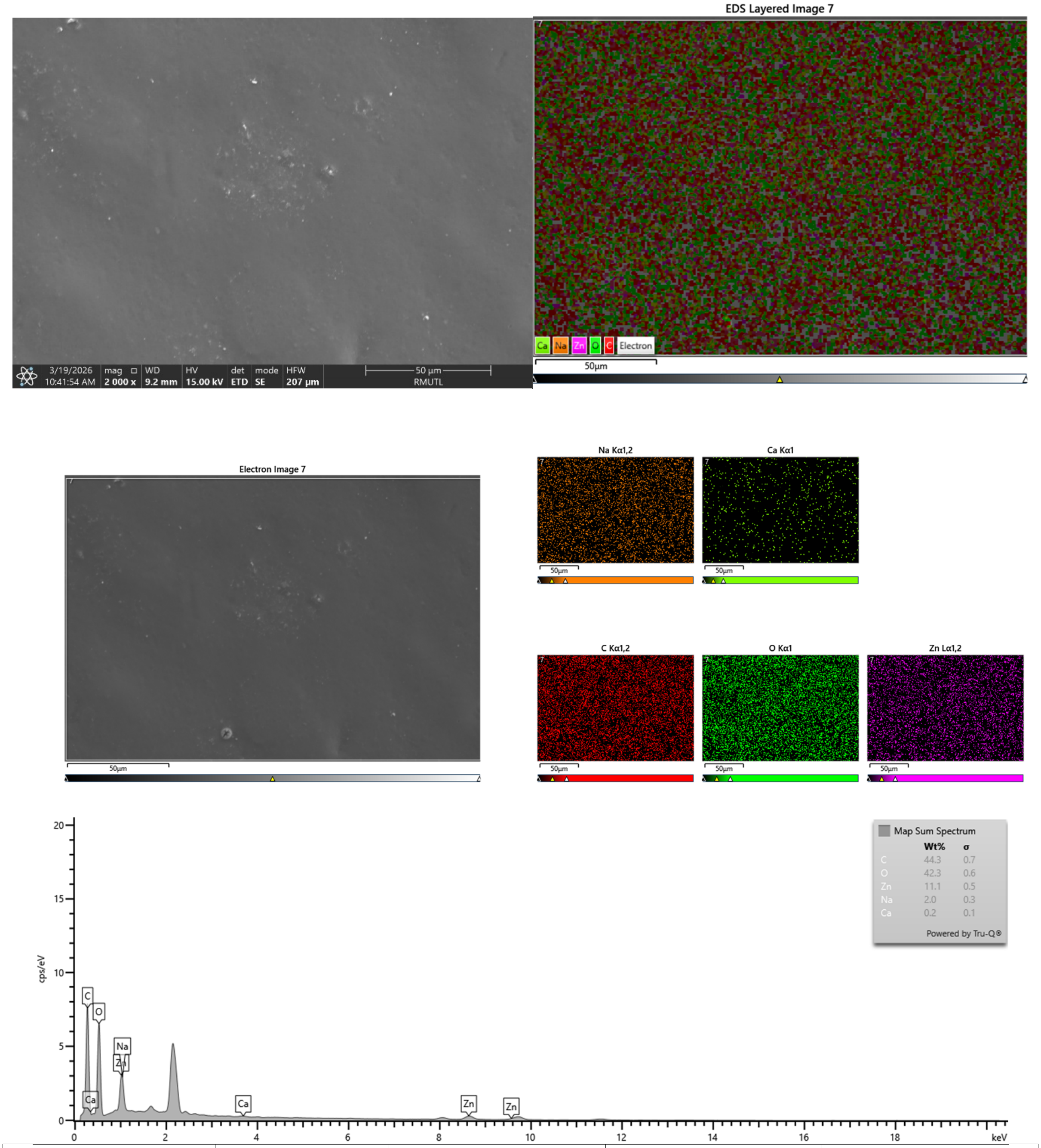

Map Sum Spectrum				
**Element**	**Line Type**	**Weight %**	**Weight % Sigma**	**Atomic %**
C	K series	44.33	0.65	55.93
O	K series	42.31	0.64	40.08
Na	K series	1.99	0.30	1.31
Ca	K series	0.24	0.13	0.09
Zn	L series	11.13	0.49	2.58
Total		100.00		100.00

Figure S8. SEM micrographs and EDX elemental maps of T0Z2 film formulations

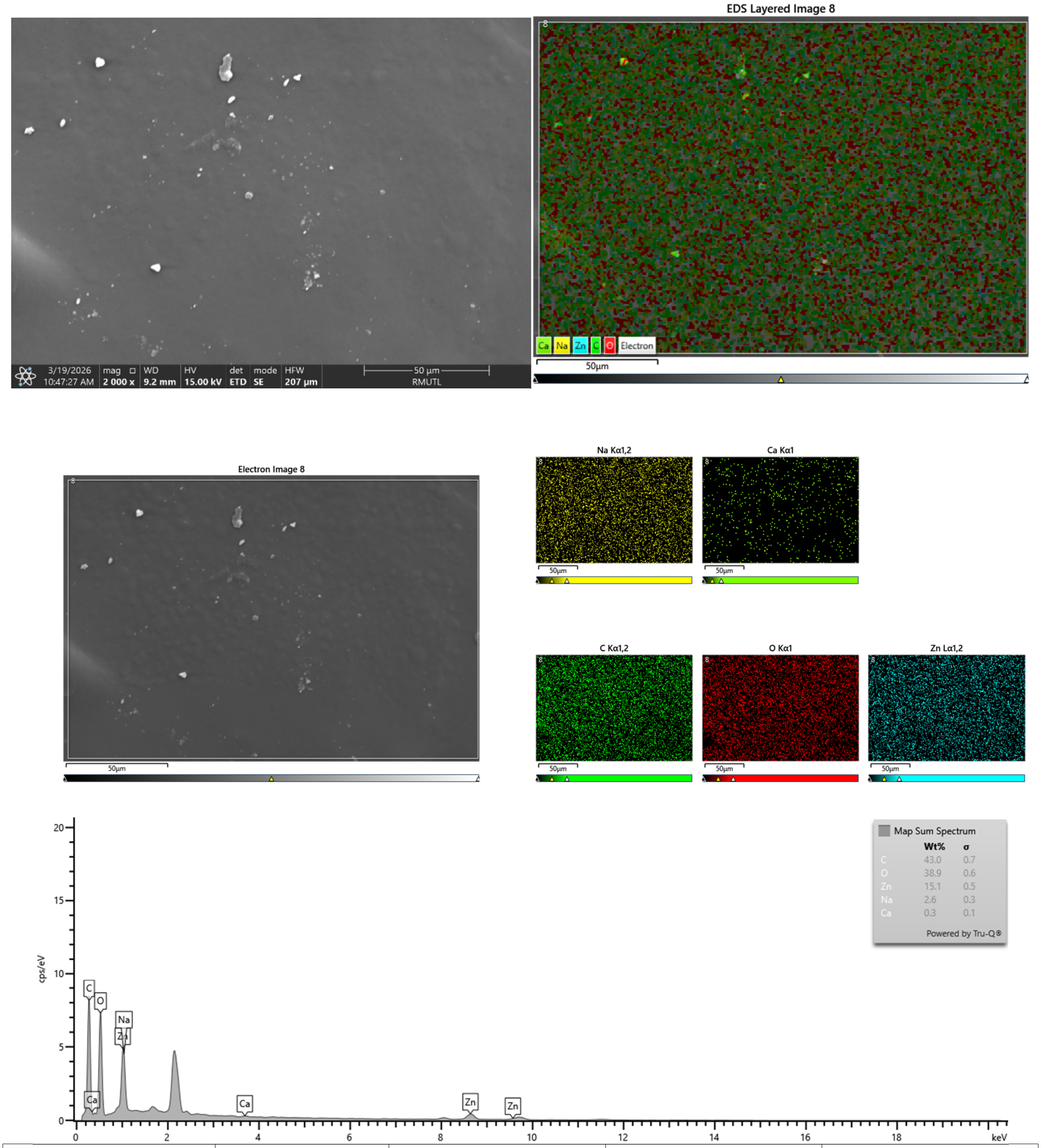

Map Sum Spectrum				
**Element**	**Line Type**	**Weight %**	**Weight % Sigma**	**Atomic %**
C	K series	43.02	0.65	56.24
O	K series	38.95	0.61	38.22
Na	K series	2.63	0.32	1.79
Ca	K series	0.33	0.11	0.13
Zn	L series	15.08	0.50	3.62
Total		100.00		100.00

Figure S9. SEM micrographs and EDX elemental maps of T0Z3 film formulations

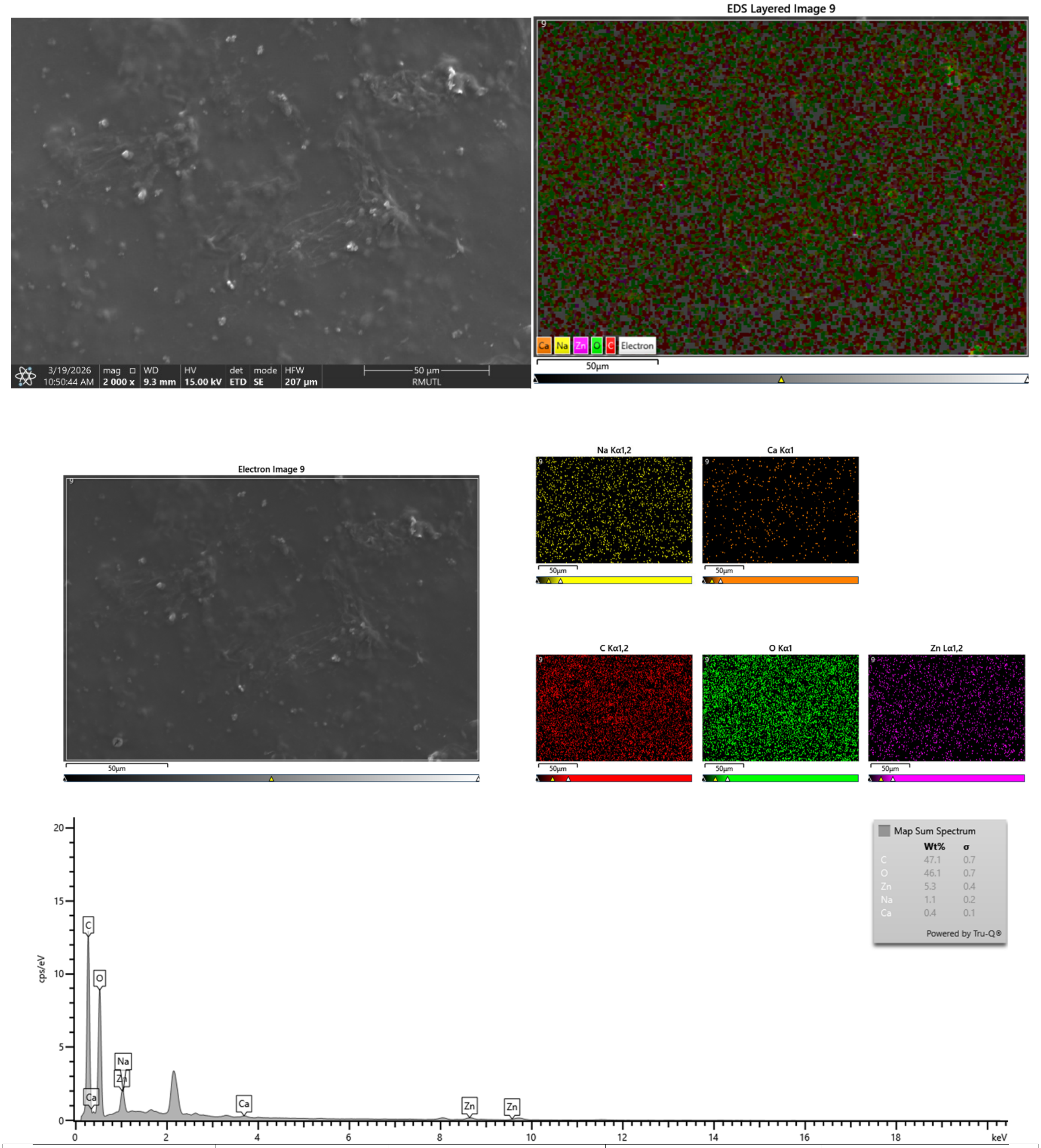

**Map Sum Spectrum**				
**Element**	**Line Type**	**Weight %**	**Weight % Sigma**	**Atomic %**
C	K series	47.05	0.67	56.45
O	K series	46.13	0.67	41.55
Na	K series	1.09	0.24	0.68
Ca	K series	0.40	0.12	0.14
Zn	L series	5.32	0.44	1.17
Total		100.00		100.00

Figure S10. SEM micrographs and EDX elemental maps of T1Z1 film formulations

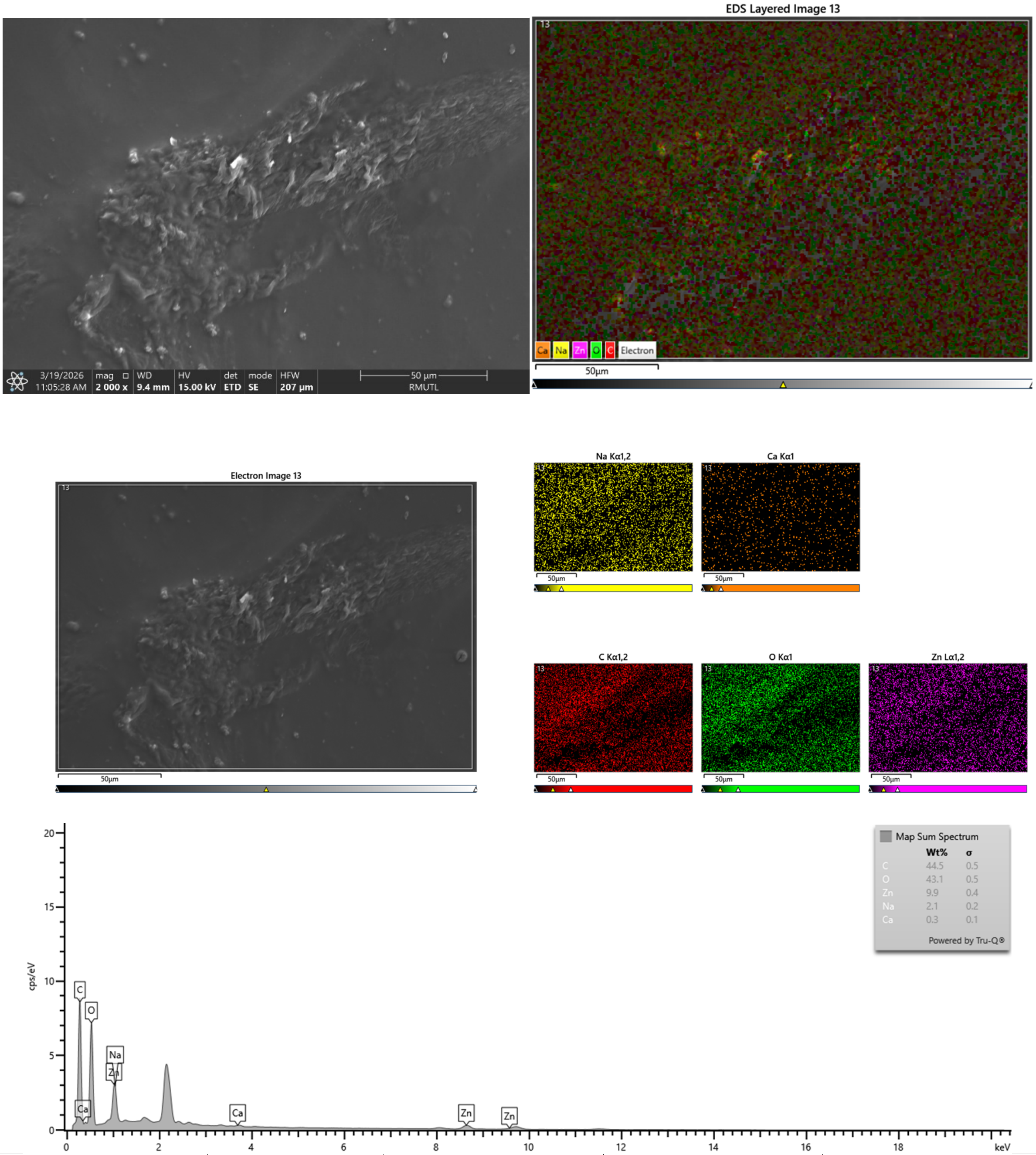

**Map Sum Spectrum**				
**Element**	**Line Type**	**Weight %**	**Weight % Sigma**	**Atomic %**
C	K series	44.52	0.54	55.72
O	K series	43.08	0.53	40.48
Na	K series	2.14	0.24	1.40
Ca	K series	0.34	0.10	0.13
Zn	L series	9.91	0.40	2.28
Total		100.00		100.00

Figure S11. SEM micrographs and EDX elemental maps of T1Z2 film formulations

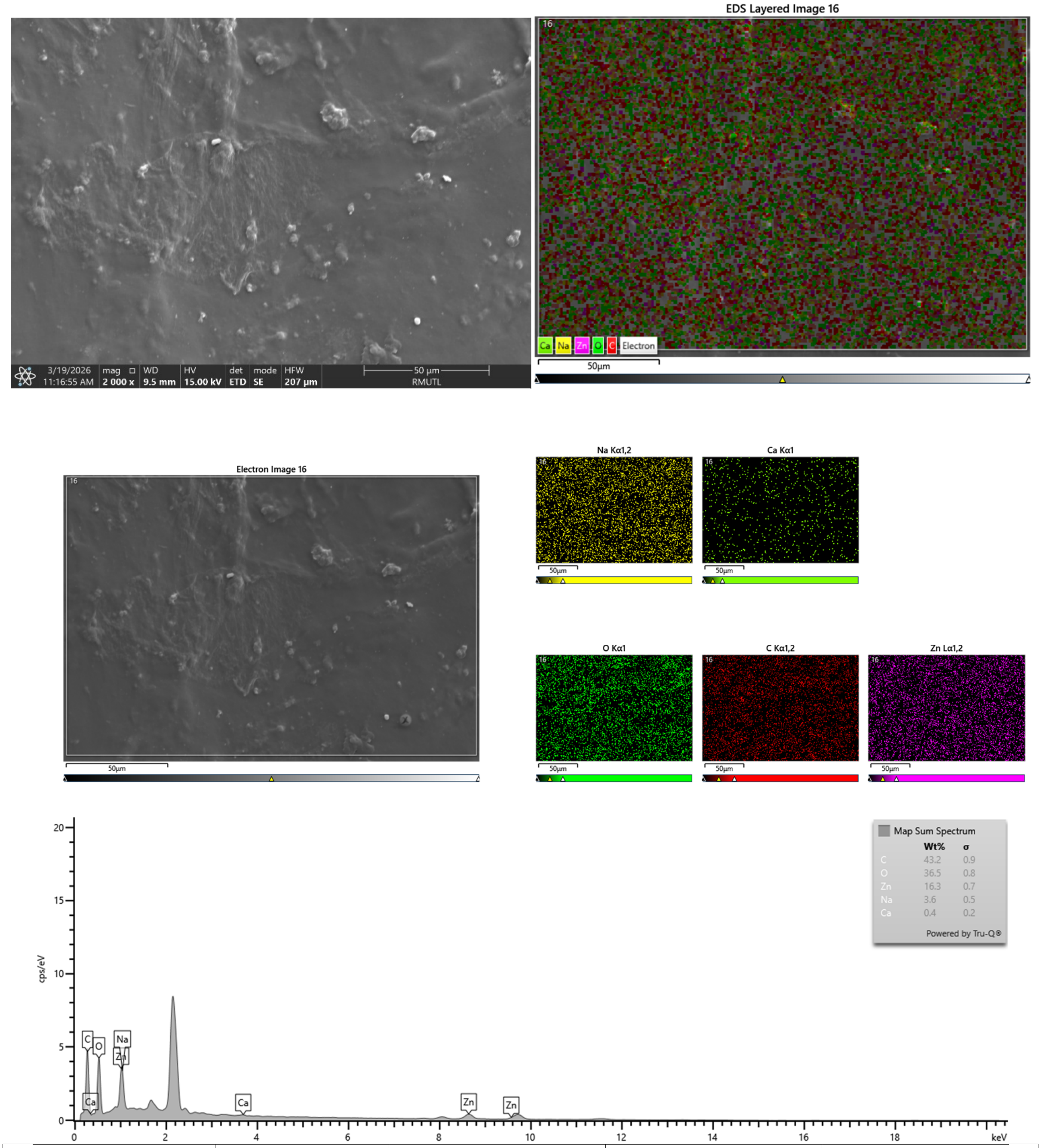

**Map Sum Spectrum**				
**Element**	**Line Type**	**Weight %**	**Weight % Sigma**	**Atomic %**
C	K series	43.17	0.89	57.11
O	K series	36.51	0.83	36.26
Na	K series	3.62	0.47	2.50
Ca	K series	0.45	0.21	0.18
Zn	L series	16.26	0.70	3.95
Total		100.00		100.00

Figure S12. SEM micrographs and EDX elemental maps of T1Z3 film formulations

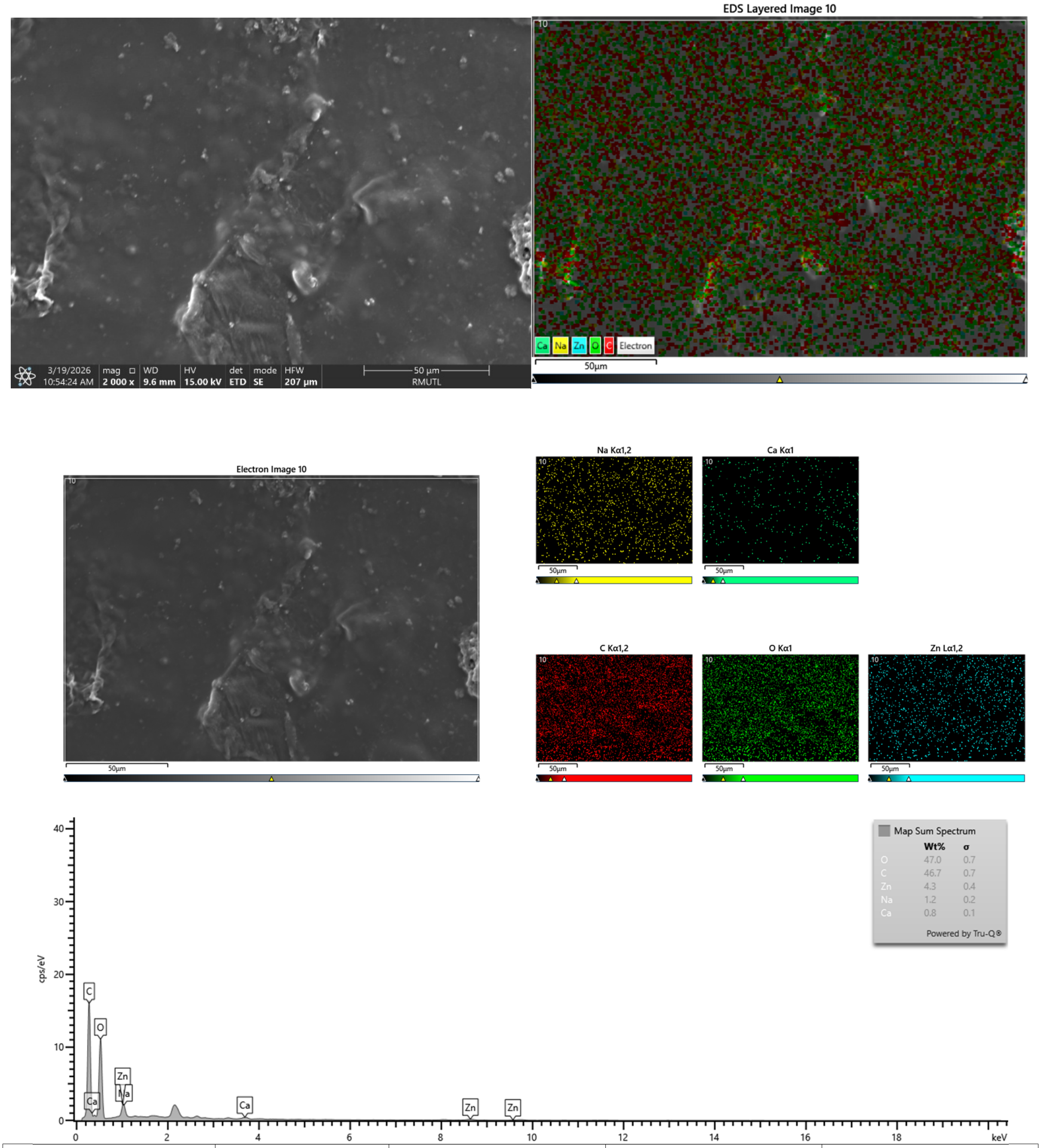

Map Sum Spectrum				
**Element**	**Line Type**	**Weight %**	**Weight % Sigma**	**Atomic %**
C	K series	46.73	0.73	55.87
O	K series	46.95	0.73	42.14
Na	K series	1.21	0.24	0.76
Ca	K series	0.82	0.13	0.30
Zn	L series	4.28	0.45	0.94
Total		100.00		100.00

Figure S13. SEM micrographs and EDX elemental maps of T2Z1 film formulations

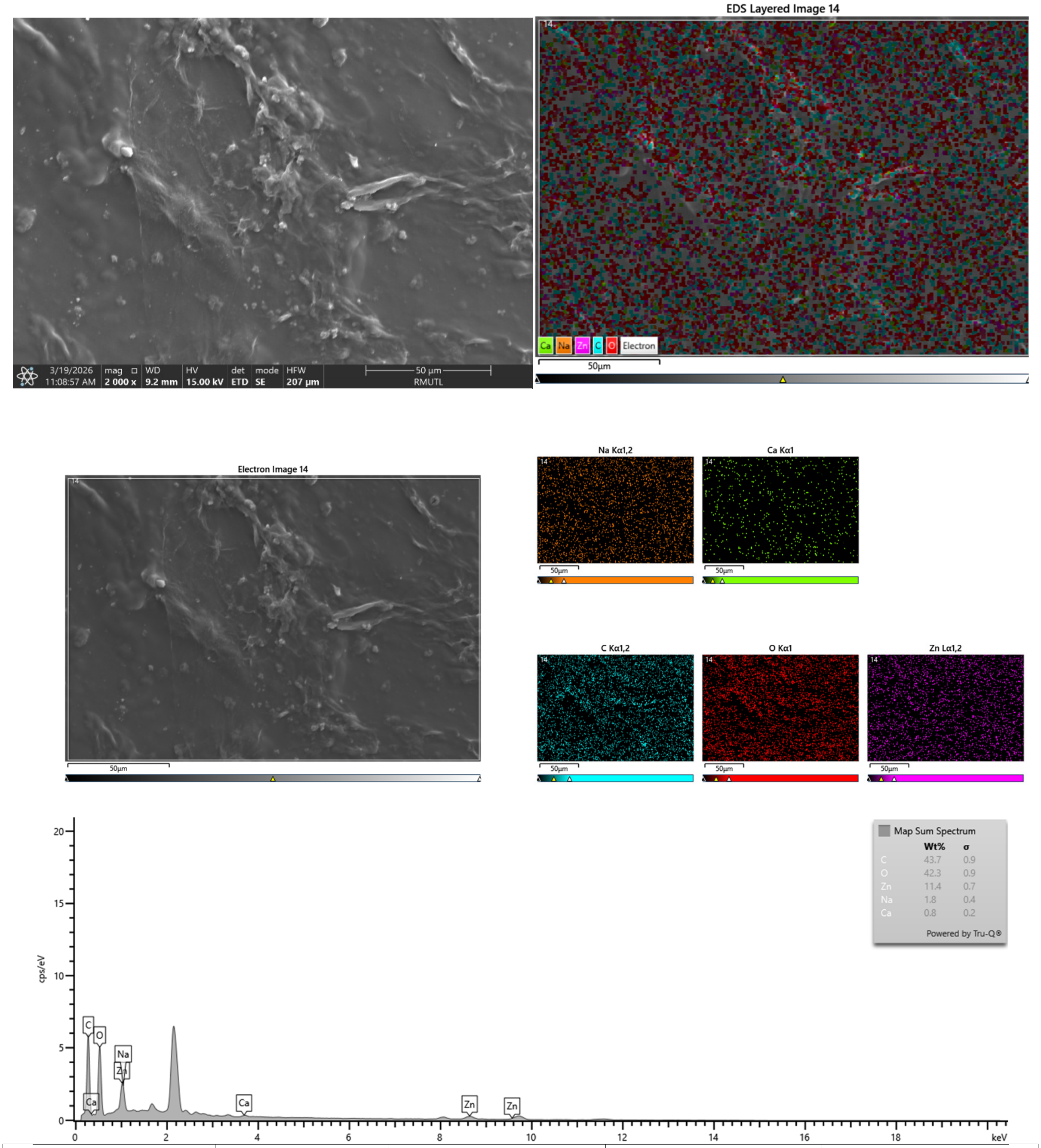

Map Sum Spectrum				
**Element**	**Line Type**	**Weight %**	**Weight % Sigma**	**Atomic %**
C	K series	43.65	0.92	55.46
O	K series	42.35	0.90	40.39
Na	K series	1.77	0.44	1.17
Ca	K series	0.79	0.21	0.30
Zn	L series	11.44	0.72	2.67
Total		100.00		100.00

Figure S14. SEM micrographs and EDX elemental maps of T2Z2 film formulations

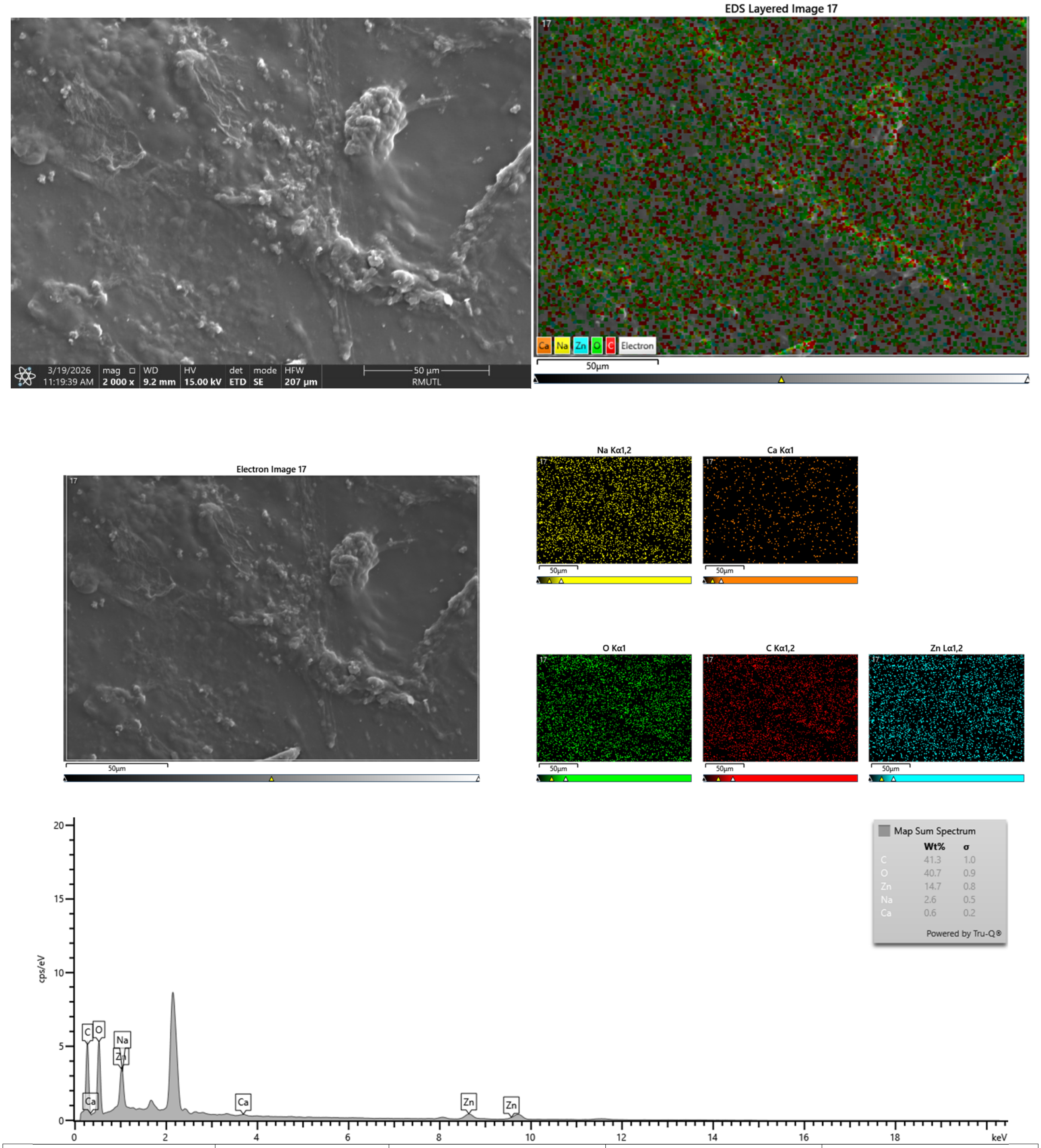

**Map Sum Spectrum**				
**Element**	**Line Type**	**Weight %**	**Weight % Sigma**	**Atomic %**
C	K series	41.34	1.00	54.29
O	K series	40.70	0.95	40.13
Na	K series	2.59	0.52	1.78
Ca	K series	0.63	0.23	0.25
Zn	L series	14.74	0.79	3.56
Total		100.00		100.00

Figure S15. SEM micrographs and EDX elemental maps of T2Z3 film formulations

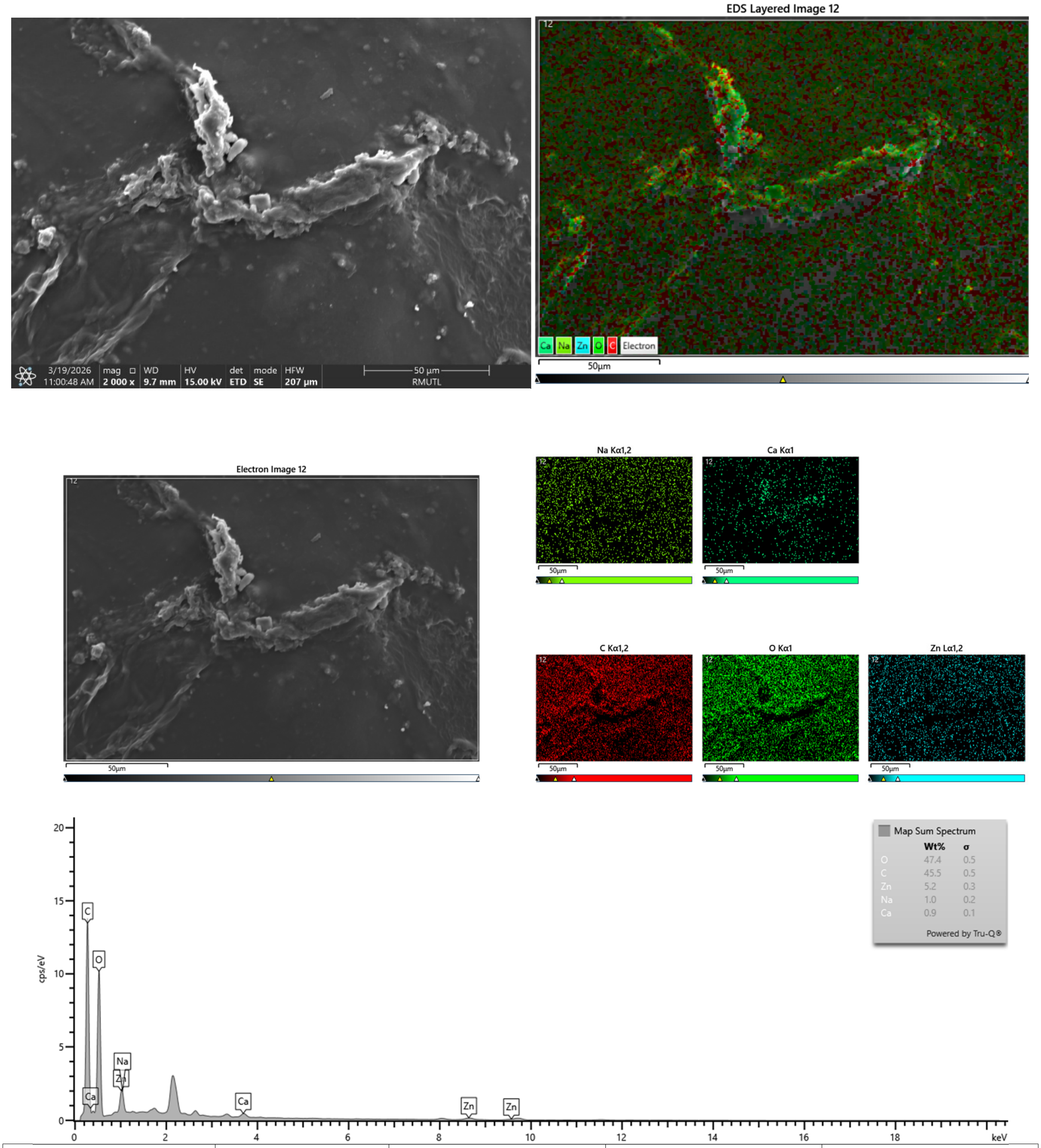

**Map Sum Spectrum**				
**Element**	**Line Type**	**Weight %**	**Weight % Sigma**	**Atomic %**
C	K series	45.48	0.49	54.90
O	K series	47.45	0.50	42.99
Na	K series	1.00	0.17	0.63
Ca	K series	0.91	0.10	0.33
Zn	L series	5.16	0.31	1.15
Total		100.00		100.00

Figure S16. SEM micrographs and EDX elemental maps of T3Z1 film formulations

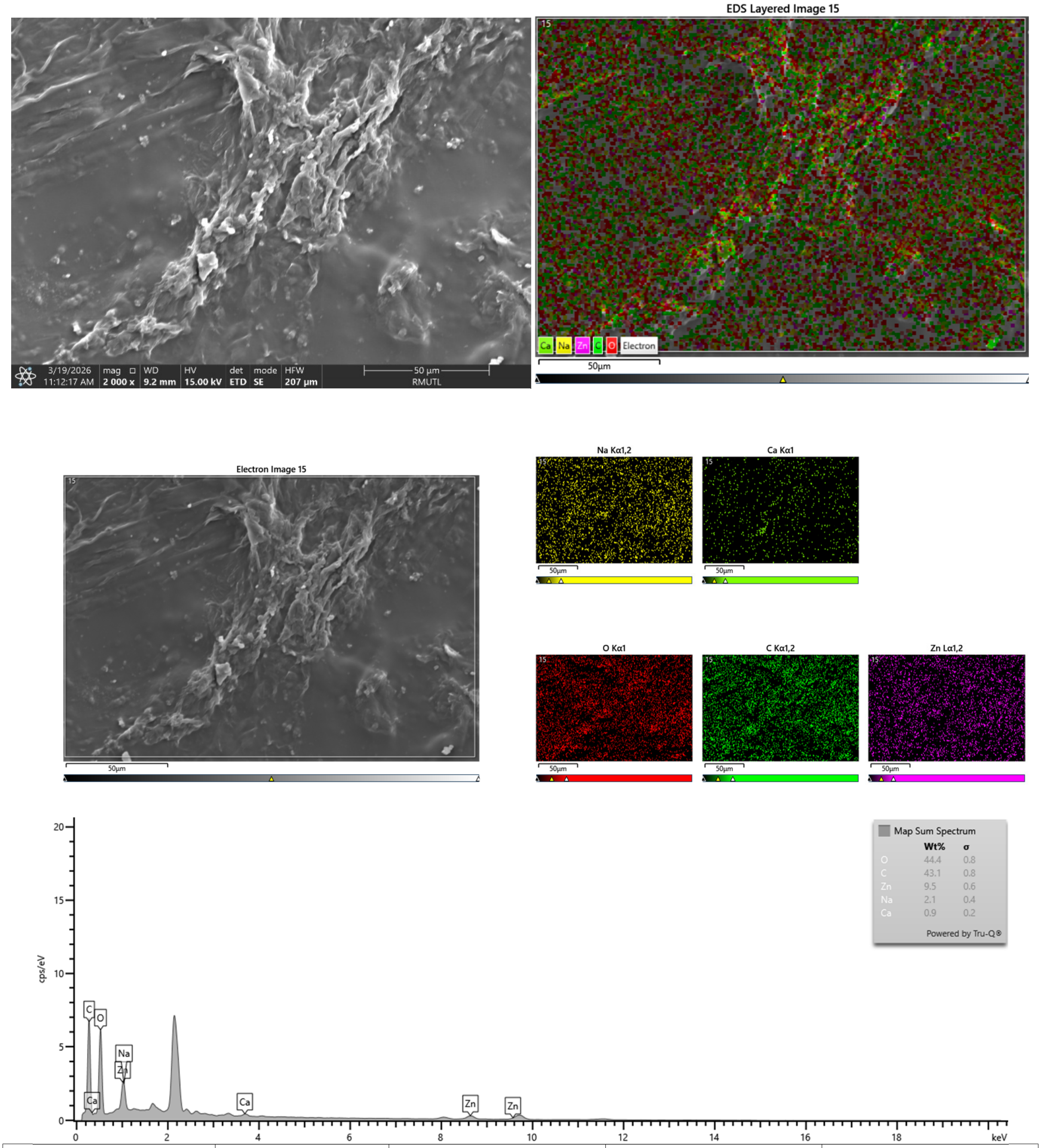

Map Sum Spectrum				
**Element**	**Line Type**	**Weight %**	**Weight % Sigma**	**Atomic %**
C	K series	43.07	0.77	54.16
O	K series	44.41	0.76	41.93
Na	K series	2.07	0.35	1.36
Ca	K series	0.93	0.18	0.35
Zn	L series	9.52	0.59	2.20
Total		100.00		100.00

Figure S17. SEM micrographs and EDX elemental maps of T3Z2 film formulations

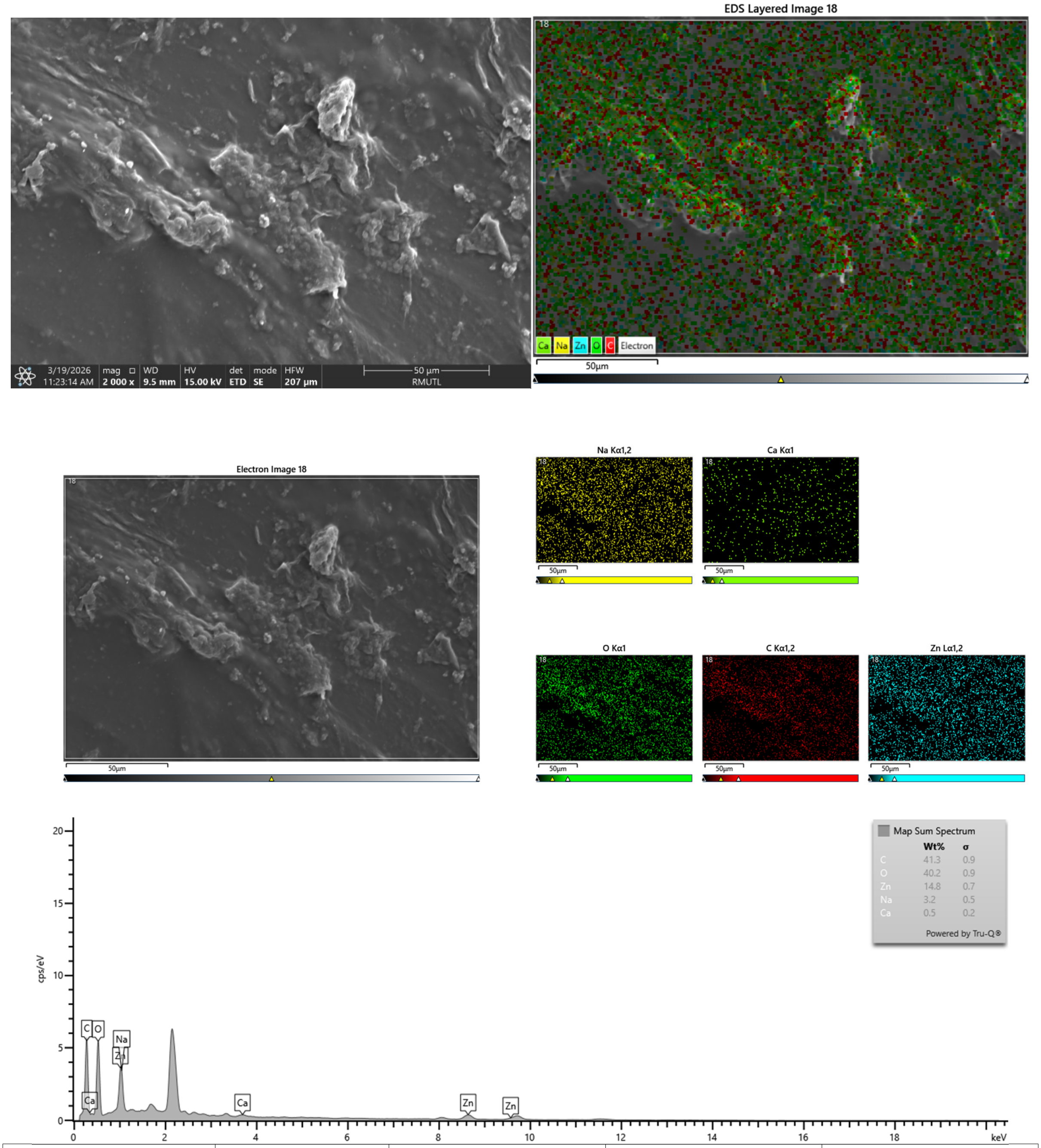

Map Sum Spectrum				
Element	Line Type	Weight %	Weight % Sigma	Atomic %
C	K series	41.29	0.92	54.32
O	K series	40.21	0.87	39.71
Na	K series	3.19	0.47	2.19
Ca	K series	0.53	0.20	0.21
Zn	L series	14.79	0.72	3.57
Total		100.00		100.00

Figure S18. SEM micrographs and EDX elemental maps of T3Z3 film formulations

3.3.4 Thermal Properties (DSC and TGA)

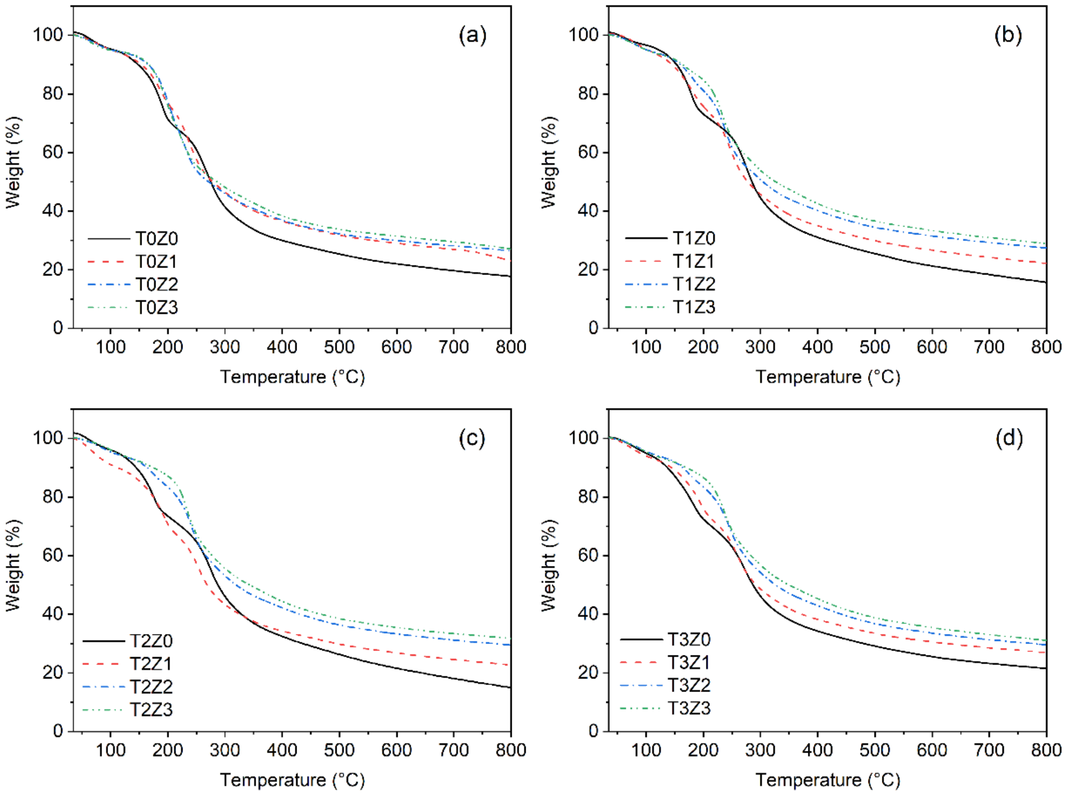

Figure S19. TGA thermograms of chitosan-based active packaging films grouped by TE concentration: (a) 0%, (b) 0.1%, (c) 0.2%, and (d) 0.3% w/v, with varying ZnO levels (0-0.3% w/v).

3.3.6 Antioxidant Activity

Table S5. Trolox equivalent antioxidant capacity (TEAC) of chitosan-based films incorporated with *Terminalia catappa* leaf extract (TE) and ZnO.

Formulation	DPPH-TEAC (mg Trolox/g film)	ABTS-TEAC (mg Trolox/g film)
T0Z0	7.2 ± 0.7^{g}	4.6 ± 0.2^{k}
T1Z0	11.5 ± 0.4^{ef}	7.5 ± 0.5^{j}
T2Z0	15.1 ± 0.3^{cd}	10.5 ± 0.5^{i}
T3Z0	18.4 ± 1.0^{b}	12.4 ± 1.1^{gh}
T0Z1	7.4 ± 0.6^{g}	5.50 ± 0.14^{k}
T0Z2	7.9 ± 1.2^{g}	5.6 ± 0.4^{k}
T0Z3	7.7 ± 1.1^{g}	5.8 ± 0.7^{k}
T1Z1	11.3 ± 1.6^{ef}	11.7 ± 0.2^{hi}
T1Z2	10.7 ± 1.4^{f}	13.9 ± 1.1^{fg}
T1Z3	14 ± 2^{de}	15.3 ± 0.6^{def}
T2Z1	16.6 ± 1.4^{bc}	15.2 ± 0.6^{ef}
T2Z2	$16 \pm 3b^{cd}$	20.7 ± 0.5^{b}
T2Z3	11.0 ± 0.7^{f}	17.0 ± 0.4^{d}
T3Z1	13.8 ± 1.9^{de}	16 ± 2^{de}
T3Z2	18 ± 2^{b}	18.8 ± 0.5^{c}
T3Z3	22.1 ± 0.2^{a}	23.9 ± 0.6^{a}

Values are mean ± SD (n = 3). Different superscript letters within a column indicate significant differences ($p < 0.05$, Tukey's HSD). TE: *T. catappa* leaf extract concentration (T0 = 0%, T1 = 0.1%, T2 = 0.2%, T3 = 0.3% w/v). ZnO concentration (Z0 = 0%, Z1 = 0.1%, Z2 = 0.2%, Z3 = 0.3% w/v).

3.3.7 Antimicrobial Activity

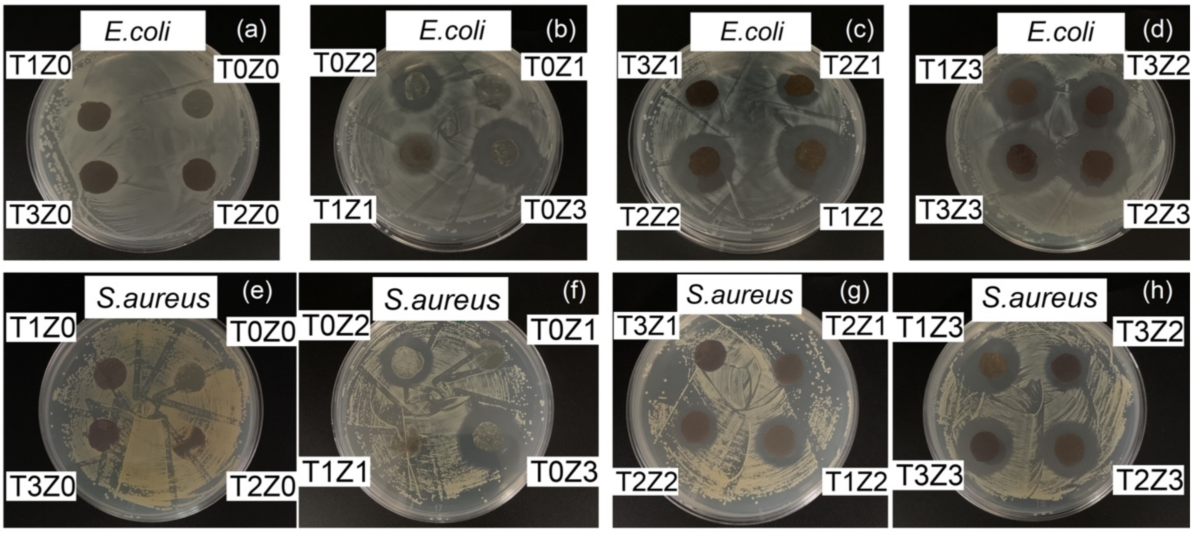

Figure S20. Photographs of disc diffusion assay plates showing inhibition zones of chitosan-based films against *E. coli* (a–d) and *S. aureus* (e–h). Film discs were incubated at 37 °C for 24 h.

UNION SCIENCE

บริษัท ยูเนี่ยน ซายน์ จำกัด (สำนักงานใหญ่)
Union Science co.,ltd. (Head Office)
257/18-19 Suthep Rd., T.Suthep, A.Muang, Chiang Mai, Thailand 50200
257/18-19 ถ.สุเทพ ต.สุเทพ อ.เมือง จ.เชียงใหม่ 50200
Tel. 053-281801-2 Line ID : @Unionscience
Website : http://www.unionscience.co.th
E-mail : sales@unionscience.biz

ALASKA CRAB CHITOSAN OLIGOMER TYPE (100MESH)

Certificate of analysis

Date : 18/10/2021

Sample name : Alaska Crab Chitosan Oligomer Type (100 mesh pass)
From . King Crab Shell
Lot number : 20211017

MFG : 17/10/2021
EXP : 16/10/2024

Chemical and Microbiological parameter

Parameter	Results	Specification
1. Viscosity	Less than 5 cps. (0.25 gram in 0.5% Acetic acid solution 50ml.)	Less than 5 cps. (0.25 gram in 0.5% Acetic acid solution 50ml.)
2. Degree of deacetylation	92.00%	More than 90%
3. Moisture	8.00%	Less than 10%
4. Ash Content	0.70%	Less than 1%
5. Heavy metal	Less than 2 ppm. (As lead ,Pb)	Less than 2 ppm. As lead (Pb)
6. Arsenic	Less than 2 ppm. (As Arsenic(III)Oxide;As_2O_3)	Less than 2 ppm. As Arsenic (III) oxide (As_2O_3)
7. Total Plate Count	Less than 1,000 cfu./g.	Less than 1,000 cfu./gram.
8. Coliform Bacteria / *E.coli*	Absent	Absent

Remark: King Crab Chitosan Powder is food grade
Scientific Name : *Paralithodes Camtschaticus*
Species : *P. Camtschaticus*
Shelf Life : 3 Years

Approved by

Mr. Kajohnsak Maneerat
Manager of Quality assurance division

สำเนาควบคุม

ISO 9001

Figure S21. Official Certificate of Analysis (COA) of the commercial food-grade chitosan oligomer (derived from king crab shell, Paralithodes camtschaticus) utilized in this study. The document, provided by the supplier (Union Science Co., Ltd., Chiang Mai, Thailand; Lot No. 20211017), corroborates the key physicochemical specifications, specifically indicating a low viscosity (< 5 cps) and a high degree of deacetylation (92.00%).

2.1.3. Determination of chitosan molecular weight

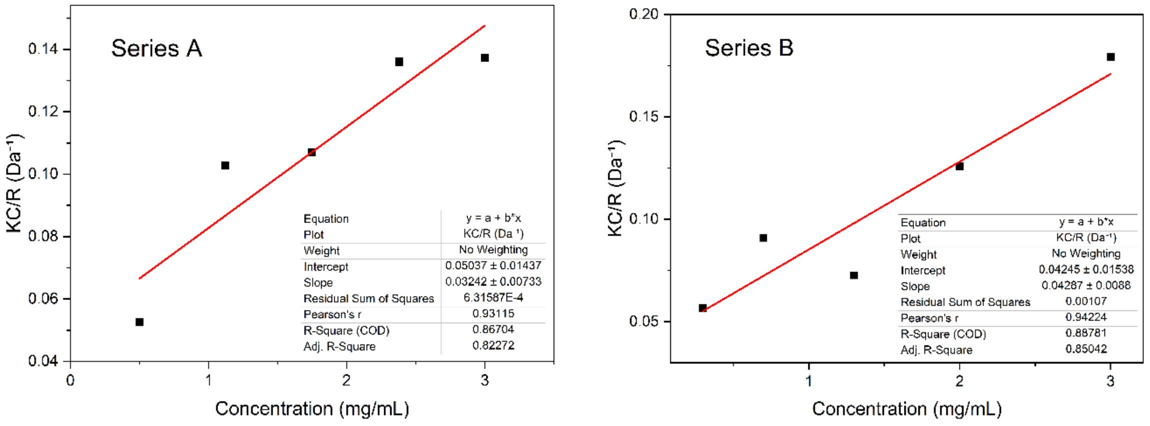

Figure S22. Debye plots of chitosan samples obtained from two independent concentration series in 0.1 M acetic acid/0.2 M NaCl at 25 °C. (A) Series A and (B) Series B.

Table S6. Summary of linear fitting of Debye plots and calculated weight-average molecular weight (Mw) of chitosan samples in 0.1 M acetic acid/0.2 M NaCl at 25 °C

Series	Concentrations (mg/mL)	Intercept (Da^{-1})	Slope (Da^{-1}·mL/mg)	Mw (kDa)	R^2
A	0.5, 1.12, 1.75, 2.38, 3.0	0.05037 ± 0.01437	0.03242 ± 0.00733	19.9	0.867
B	0.3, 0.7, 1.3, 2.0, 3.0	0.04245 ± 0.01538	0.04287 ± 0.00880	23.6	0.888
